# Supplementary material for: Real-world stress resilience is associated with the responsivity of the locus coeruleus
Source: Nat Commun. 2021 Apr 15;12:2275. doi: 10.1038/s41467-021-22509-1 (PMC8050280; doi:10.1038/s41467-021-22509-1)
Supplement: Supplementary file 1 — Supplementary Information [file 41467_2021_22509_MOESM1_ESM.pdf]

Supplemental Information

for

Real-world stress resilience is associated with the responsivity of the locus coeruleus

Marcus Grueschow[1]*, Nico Stenz[2,3], Hanna Thörn[2,3,4], Ulrike Ehlert[4], Jan Breckwoldt[5], Monika Brodmann Maeder[6], Aristomenis K. Exadaktylos[6], Roland Bingisser[7], Christian C. Ruff[1#], Birgit Kleim[2,3#]*

[1] Zurich Center for Neuroeconomics (ZNE), Department of Economics, University of Zurich, Zurich, Switzerland

[2] Division of Experimental Psychopathology and Psychotherapy, Dept of Psychology, University of Zurich, Zurich, Switzerland

[3] Department of Psychiatry, Psychotherapy and Psychosomatics, University of Zurich, Zurich, Switzerland

[4] Division of Clinical Psychology and Psychotherapy, Dept of Psychology, University of Zurich, Zurich, Switzerland

[5] Medical School, Deanery, University of Zurich, Zurich, Switzerland

[6] Accident and Emergency Department, Inselspital Bern, Bern, Switzerland

[7]Department of Emergency Medicine, University Hospital Basel, Basel, Switzerland

Supplemental Methods

Participant exclusion criteria

Forty of the 94 medical students initially expressing interest to participate in the study had to be excluded due to the following a-priori criteria: Insufficient German skills, self-reported psychopathology (including depression and anxiety), a scheduled internship in an area with projected low stress exposure (e.g., dermatology), or fMRI safety exclusion criteria (pacemaker or neurostimulator, hearing aid, insulin or pain pump, implants like cochlea implants or prostheses with metallic parts, irremovable ferromagnetic material on or in the body like piercings, metal splitter injuries, metal clips). Additional exclusion criteria constituted claustrophobia, inability to lie in the MRI scanner due to tremor or coughing, recent (<6 months) unhealed tattoo, and pregnancy. Moreover, six participants canceled their participation or did not appear at the appointed time for the baseline interview.

Questionnaires:

State-Trait Anxiety Inventory (STAI):

Anxiety symptoms [1] were assessed with the State-Trait Anxiety Inventory (STAI) [2]. The trait anxiety scale consists of 20 items that each measures the intensity of anxiety. Each subscale ranges from 20-80, with higher scores indicating greater anxiety levels. Trait anxiety was indexed at all three time points (t_0, t_1, t_2) to investigate potential changes in anxiety severity during the internship. Internal consistency in the present study was high, Cronbach's $\alpha = 0.90$.

Patient health questionnaire (PHQ):

Depression symptom severity was assessed with the Patient Health Questionnaire (PHQ-9)[3], a self-administered version of the PRIME-MD instrument for common mental disorders. Scoring of each of the nine DSM-IV depression criteria results in a total PHQ-9 score ranging from 0

to 27, with a cut-off score of 5 for mild depression[4]. Depression was indexed at all three time points (t_0, t_1, t_2) to investigate potential changes in depression severity during the internship. Internal consistency in the present study was good, Cronbach's $\alpha = 0.80$.

An adapted version of the **Trauma Checklist** derived from the Posttraumatic Diagnostic Scale (PDS)[5] was used to index individual ***pre-trauma-scores*** by summing the number of potentially traumatic events encountered at baseline, personally or as a witness, for each participant. Participants reported yes/no regarding the following types of incidents: accident, fire or explosion, natural disaster, violent assault, sexual assault, combat in war or residence in a warzone, captivity, imprisonment, hostage taking, torture, live threatening disease or other types of potentially traumatic events. The level of prior stressful life events correlated neither with LC-responsivity ($p= 0.2640$, $R=0.1644$) nor with LC-Amygdala connectivity ($p= 0.9820$, $R= 0.0033$). In addition, there was also no significant correlation between number of previously experienced traumatic events and mean anxiety or depression symptom changes ($p= 0.7671$, $R= -0.0439$). While such relationships might potentially be expected in patients suffering from anxiety and depression well above clinically-relevant thresholds, they may not be present in our specific cohort of well-adapted medical students with relatively small numbers of prior traumatic events (mean number of pre-trauma events: 1.1, range: 0-4).

Adverse events during internship.

We conducted a control analysis, testing how individual symptom severity changes may relate to the exposure to potentially stress-inducing events during the internship. We did this by having participants indicate how often one of the following adverse types of incidents occurred: death of a patient, a particularly invasive treatment, grieving or agitated relatives, a treatment during which the participant or a colleague made a severe mistake, and similarly adverse events

(13 items, adapted for the present study from Weiss et al., 2010). The experienced severity of these events was individually quantified on a scale ranging from 1 (not stressful) to 4 (extremely stressful). In order to derive an individual final score for adverse events, we multiplied their number and severity for each participant and correlated this measure with observed individual symptom changes.

This revealed no significant relationship between stressful event exposure during the internship and mean depression symptom changes (Pearson-correlation p=0.346; R=-0.139). However, we did find that mean anxiety symptoms increased significantly with experienced adverse events (Pearson-correlation p=0.006; R=-0.393, see supplemental methods above for details). We tested whether this explained any additional variance above and beyond our original prospective predictors, by including adverse events in our original full model. This showed that adverse events did not explain any substantial variance (R^2) or adjusted variance (adj R^2), while both LC and LC-Amygdala connectivity remained strong predictors of anxiety symptom changes (See supplemental table 4). These findings also did not depend on the choice of LC-mask (1SD or 2SD).

Stimulus presentation

All stimuli were displayed on a grey projection screen (using the Cogent2000-toolbox, http://www.vislab.ucl.ac.uk/cogent_2000.php, implemented in Matlab, The MathWorks, Inc., Natick, Massachusetts, United States) that participants viewed by means of a mirror system mounted atop the MR head coil. The task comprised 50 congruent, 50 incongruent, and 20 neutral (grey display without a face) trials, presented in pseudorandom order and counterbalanced for equal numbers of congruent-congruent (CC), congruent-incongruent (CI), incongruent-congruent (IC), and incongruent-incongruent (II) stimulus pairings. To avoid any priming effects, there were neither direct repetitions of the same face with varying word

distracters nor direct repetitions of exact face-word-distracter combinations[6,7]. Genders, identities, and affective expressions on the faces were randomized throughout the task and stimulus occurrences were counterbalanced across trial types and response buttons. Subjects were instructed to respond as fast as possible to the stimulus by pressing one of two buttons (left: happy, right: fear or vice-versa) on an MR-compatible response box, while trying to maintain high accuracy.

Data analysis

Behavioral analyses.

We adopted identical behavioral analysis procedures as the previous work investigating behavioral and neural conflict processing [8-10]. Behavioral data consisted of both reaction times (excluding error and post-error trials) and accuracy rates. A response was considered correct when the emotional valence of the face expression was correctly identified. Trials with response times above 2 standard deviations from the mean (across all trials) were excluded from analysis (and regarded as trials of no interest in the fMRI-models[8-10], see below). 45 out of 48 Participants viewed 200 trials (2 runs, 100 trials per run). Some subjects missed a few trials and 3 subjects did only 1 run (100 trials). Since we removed trials with an RT larger than 2 standard deviations above the mean, the median trial number was 190 trials (minimum: 94, maximum: 196 trials). For the congruency-sequence-effect analysis, we mean-centered the RT-data for each individual to focus on within-subject variability of response times.

fMRI image acquisition.

Subjects performed two fMRI sessions of the emotional stroop task, each lasting 9.75 minutes. During each session, we acquired 225 T2*-weighted whole-brain echo planar images using a Philips Achieva 3 T whole-body scanner (Philips Medical Systems, Best, The Netherlands)

equipped with an 8-channel Philips sensitivity-encoded (SENSE) head coil. Imaging parameters were: 2600 ms repetition time (TR); 37 slices (transversal, ascending acquisition); 2.6 mm slice thickness; 2.5 mm x 2.5 mm in-plane resolution; 0.65 mm gap; 90° flip angle. To measure at fully equilibrated magnetic field, five dummy-image excitations were performed and discarded before functional image acquisition started. Additionally, we acquired a high-resolution T1-weighted 3D fast-field echo structural scan used for image registration during post-processing (sequence parameters: 181 sagittal slices; matrix size: 256 x 256; voxel size: 1 x 1 x 1 mm; TR/TE/TI: 8.3/2.26/181 ms).

fMRI image pre-processing.

Image preprocessing and analysis were conducted using SPM8 (Wellcome Trust Centre for Neuroimaging). Functional images were slice-time corrected (to the middle slice acquisition time) and realigned (accounting for subjects' head motion). Each subjects' T1-weighted structural image was co-registered to the mean functional image and normalized to the standard T1-MNI template using the "Unified Segment" procedure provided by SPM8 [11]. The procedure incorporates spatial normalization and tissue class segmentation within the same model so that an optimal solution is found for both within the same framework. The procedure uses 6 tissue probability classes for MW, GM, CSF, skull, soft tissue and other (i.e.: eyes). These standardized probability maps for different tissue classes were constructed from a large number of brains that are registered into a common space. In the 'unified segment' Bayesian framework, these maps represent the prior probability of any voxel belonging to a particular tissue class (priors). The procedure warps the standard tissue probability maps to match the current subjects' maps by maximizing their mutual information. The inverse transform can then be used to normalize the functional images to standard MNI space. A recent report stated that when taking prior tissue class information into account, the 'unified segment' approach as

implemented in SPM outperforms several other methods in both precision of registration as well as tissue classification [12]. The functional images were normalized to the standard MNI template using this transformation, spatially resampled to 2.5 mm isotropic voxels, and smoothed using a Gaussian kernel (FWHM, 6mm).

Eye measures.

During scanning, eye movements were sampled at 250Hz using an MR-compatible infrared EyeLink II CL v4.51 eye-tracker system (SR Research Ltd.). In order to account for the effects of eye movements and blinks on BOLD responses, we added them as regressors of no interest to the general linear model (see below). Saccades were defined as eye movements larger than 0.5 degrees visual angle [13]. Blinks were defined as periods of signal loss lasting longer than 80 ms and shorter than 2000 ms [14]; these epochs were removed from the pupil data and filled in by linear interpolation.

Pupil dilation cluster-correction.

To identify time windows during which the pupil dilation significantly differs between relevant trial types (I>C, CI>II, IC>CC, **Figure 6**), while avoiding false positive clusters, we applied Bonferroni-correction [15,16] via a cluster-based permutation test following [17]. We used a cluster-forming threshold of T = 2.02 corresponding to a two-sided p-value of 0.05 given 47 degrees of freedom (N=48). The procedure first calculates the one-sample t-statistic across all participants' average difference between relevant contrasts for each 4-millisecond bins in the pupil dilation time series. Next, the size of continuous temporal clusters, defined as the number of adjacent time bins exceeding the cluster-forming threshold were identified and tested against cluster sizes observed by chance. To this end, a null distribution of cluster sizes was generated by permuting the labels for each trial and time bin within-participant by flipping the sign of

each time bin randomly 1000 times and recomputing the t-statistic across all time bins for each iteration. On each iteration the *largest* permuted temporal cluster was identified and stored in the null distribution. A cluster corrected p-value is computed by dividing the number of clusters in the null distribution exceeding the number of clusters in the data by the number of iterations.

fMRI data-analysis.

We estimated a general linear model (GLM) to identify regions associated with arousal upregulation, defined as the response difference between CI and II trials. This contrast was used to test whether LC-NE arousal system responsivity predict individual stress resilience. The GLM contained four indicator functions placed at the onset of each of the possible trial types, based on current and previous trial congruency (CI, II, IC, & CC). For instance, CI is an incongruent trial (I) preceded by a congruent trial (C) while II represents an incongruent trial (I) preceded by an incongruent trial (I), and so forth. An additional indicator function modelled the onsets for trials of no interest, which included: the first trial of each session that cannot be classified with respect to preceding trial type, trials with reaction times 2 standard deviations above the participant's overall mean response time, as well as error and post-error trials that may be associated with error-related cognitive processing [9,10].

Six motion parameters (obtained during the realignment procedure) were also included as regressors of no interest to account for participants' head motion. Furthermore, we included additional regressors that accounted for variance induced by eye-related variables (blinks and saccades), to ensure that neural conflict responses and stress-resilience predictions are not confounded by these variables [18]. The model thus included additional indicator functions for the onsets of blinks and saccades.

First-level summary statistics were obtained by calculating the single-subject voxel-wise contrasts of incongruent>congruent trials (I>C, quantifying conflict, **Supplemental Figure 1**),

CI>II trials (quantifying upregulation, **Figure 1** and **supplemental Figure 2**) as well as II>CI trials (quantifying conflict adaptation, **supplemental Figure 3**). Statistical inference was performed with a random-effects General Linear Model and cluster-level inference based on non-parametric permutation tests and pseudo t-statistics for independent observations within the SnPM framework (http://warwick.ac.uk/snpm). The whole-brain FWE-corrected statistical threshold was set to $P < 0.05$ with an initial cluster-defining voxel-level threshold of T = 3.275 (equivalent to uncorrected $P < 0.001$)[17,19]. For hypothesis-guided ROI analysis of the LC-NE arousal system, we applied the identical non-parametric statistical procedure as above restricted to a 2SD-locus coeruleus volume mask [20]. To test for regions previously associated with the CI>II contrast in earlier seminal studies [9,10] (**Supplemental Figure 2-3**), amygdala ROIs were taken from the Harvard-Oxford cortical and subcortical structural atlases as used in the FSL package (https://fsl.fmrib.ox.ac.uk/fsl/fslwiki/Atlases). DLPFC regions of interest associated with the conflict response (CI>II) were created using 15mm spheres around coordinates provided by Etkin et al. 2006, and DLPFC regions of interest associated with conflict adaptation were created using 15mm sphere around coordinates provided by Muhle-Karbe, et al. 2017.

Supplemental Results

Behavior and fMRI data confirm conflict induction and congruency sequence effects

Individuals are generally slower to respond to incongruent trials [21], a finding we replicated here in the medical student cohort. Comparing incongruent with congruent trials, we found significantly increased RTs ($T_{47} = 9.88$, $p = 4.67 * 10^{-13}$, **Supplemental Figure 1C**) and decreased accuracy ($T_{47} = -5.25$, $p = 3.65 * 10^{-6}$, **Supplemental Figure 1D**). Theoretical frameworks[22] and empirical findings[10,23,24] predict that that the monitoring and processing of conflict is reflected in activity of the dorsomedial prefrontal cortex (DMPFC) and anterior

cingulate cortex (ACC). Contrasting incongruent > congruent trials (I>C), we also replicated these classic neural findings (please see **Supplemental Figure 1A-B**). Finally, we also replicated the well-documented congruency-sequence effect[9,10] (CSE) in reaction times (interaction between current and previous trial type: *beta* = -2.46, T_{47} = -2.10, p = 0.041, **Supplemental Figure 1E**), even when controlling for emotional valence (*beta* = -11.70, T_{47} = -6.06, $p < 0.001$)

Locus Coeruleus responsivity is a robust and reliable bio-marker for stress resilience

In a final analysis we attempt to quantify the usefulness of the identified bio-markers in predicting stress resilience by asking two questions: First, how much more variance can be explained by our identified biological markers compared to the current gold standard (self-report surveys assessing either past potentially traumatic experiences or the current symptom level status quo)? To this end we used a multiple GLM-approach by comparing the predictive power of a base model (containing only the scores from the traditionally used clinical resources, i.e.: respective symptom severity survey at T_0 and a survey assessing prior experience of adverse and potentially traumatic events) to increasingly more complex models, by adding our behavioral and physiological variables of interest as defined above (classic RT congruency sequence effect (CSE), LC upregulation response (LC), Pupil dilation distance (pupil), and LC-amygdala functional coupling during upregulation (LC-connectivity)). Secondly, we ask, which are the most parsimonious parameter combinations to predict individual anxiety or depression symptom change? To this end we use a stepwise-regression approach (**Methods**). For a comprehensive list of parameter test-statistics, goodness-of-fit measures and model comparisons please see **Supplemental Tables S7 and S8** using the weighted average LC-1SD mask from the physio-corrected, unsmoothed fMRI data as well as tables **S9** and **S10** for data without these corrections regarding anxiety and depression symptom changes respectively.

Lastly, we assess the out-of-sample prediction accuracy between the base model, full model (containing all parameters) and most parsimonious model using LTSO (**Methods**). Here we report the statistics for the data without optimizing brainstem imaging corrections (**tables S9 and S10**), while the main text reports these results using the weighted average LC-1SD mask from the physio-corrected, unsmoothed fMRI data.

We found that the base model, representing the current gold standard for assessing acute anxiety symptomology, was not a reliable predictor for anxiety symptom severity changes. Neither of the two self-report scores were significant ($p<0.05$) nor did this model predict prospective resilience above chance; it only explained 4% of the adjusted variance. In contrast, our identified bio-marker substantially improved predictions of anxiety symptom changes. The adjusted explained variance was increased by 400% and 300% respectively when adding either LC ($p=0.017$) or pupil ($p=0.039$) separately. The classic behavioral CSE score was neither significant on his own ($p>0.1$, model 2) nor in models containing either LC (model 3) or pupil (model 4). Having both LC and pupil regressors compete in explaining anxiety changes (model 5) further increases adjusted variance explained (effectively 20%) and establishes LC as reliable predictor ($p=0.041$) while, potentially due to shared variance, pupil becomes less reliable ($p=0.09$). Importantly, adding the individual connectivity strength between LC and amygdala during the upregulation response (model 6) leads to a massive increase in adjusted explained variance (effectively 50%, approximately 12 times the base model) and significantly above chance out-of-sample predictions ($p<0.001$, 61.8%). These results establish noradrenergic responsivity in the LC ($p=0.034$) and LC-amygdala-connectivity ($p<0.001$) during upregulation as important biological markers for anxiety symptom changes and thus stress resilience. Both these variables were also identified as major contributors in the most parsimonious model, which contained LC-connectivity ($p<0.001$), LC ($p=0.007$), and CES ($p<0.01$). This model delivered the highest adjusted explained variance of 51% and predicted

symptom severity change out-of-sample ($p<0.001$, 62.6%) despite only containing 3 parameters.

The LC conflict response is also the most reliable predictor for depression symptom severity changes, even though the base model already explained 23.3% adjusted variance, primarily driven by the PHQ-depression score at T_0 ($p=0.0002$, model 1). However, it has to be noted that PHQ at T_0 was inversely related to symptom changes, suggesting a ceiling effect or regression to the mean. Participants with increased depression scores prior to the medical internship were unlikely to increase their depression symptoms further due to real-world stress. Nevertheless, irrespective of model complexity, the individual LC upregulation response was the only biological marker that reliably related to depression symptom changes (at least $p < 0.04$), despite controlling for behavioral CSE ($p>0.74$), pupil distance ($p>0.14$), or LC-connectivity ($p=0.57$). The LC upregulation regressor added 7% of adjusted variance (30.3%, model 3) to that achieved by the base model, which was similar to that achieved by the full model including all parameters (29.7%, model 6, with 65.5% out-of-sample accuracy). LC was also the only biological marker ($p=0.01$) identified in the most parsimonious model along with the PHQ score at T_0 ($p=0.004$). This model explained 33.3% adjusted variance and significantly predicted mean symptom severity changes out-of-sample ($p<0.001$, 68% accuracy). Taken together, the comprehensive regression analyses for anxiety and depression symptom changes establish that the noradrenergic LC responsivity is a much better prospective predictor of individual stress resilience than the currently used clinical measures.

Supplemental Tables

Supplemental Table S1: Upregulation regions (CI>II)

Region	Peak-Side	Cluster Size	x	y	z		T value	p-value
**SnPM whole-brain**								
STC	L	190	-67	-32	23		5.12	0.031
Midbrain-cluster	R/L	170	6	-27	-10		4.58	0.039
PCC	R/L	155	-7	-37	33		4.30	0.042
Ant. visual cortex	R/L	130	16	-65	8		3.45	0.052
**SnPM SVC**								
LC	R		6	-37	-28		3.56	0.003^{SVC}
**Classic SVC**								
DLPFC	L		-45	21	23		3.56	0.006^{SVC}
Amygdala	L		-27	1	-33		3.6	0.045^{SVC}
LC	L		-5	-37	-28		3.55	0.016^{SVC}
LC	R		6	-37	-28		3.56	0.003^{SVC}

Upregulation regions (CI>II).

This table shows the regions identified with the contrast CI>II relating to upregulation. All p-values are FWE-corrected for a cluster-level threshhold of $p<0.05$ (generated with an initial cluster-forming threshold of $T_{(1,47)}=3.277$, equivalent to peak-level $p=0.001$). SVC indicates peak-level small-volume correction. Small-volume correction for the locus coeruleus was performed using the 2SD-mask from Keren et al.[20]. LC small-volume correction was also significant when tested with non-parametric statistics as indicated by SnPM SVC. Amygdala ROIs are taken from the Harvard-Oxford cortical and subcortical structural atlases as used in the FSL package (https://fsl.fmrib.ox.ac.uk/fsl/fslwiki/Atlases). A previous region identified with CI>II contrast is the DLPFC. Regions of interest were created using 5mm radius spheres around coordinates provided by Etkin et al. 2006. Right DLPFC: XYZ = 42/14/32; left DLPFC: XYZ = -44/18/24. Note that the right amygdala and right DLPFC failed significance for the contrast (CI>II, Amygdala: XYZ = 21/3/-30, T=2.90, Z=2.77, $p^{SVC} = 0.214$, $p_{uncorr}= 0.003$, rDLPFC: XYZ = 43/13/28, T=1.82, Z=1.79, $p^{SVC} = 0.176$, $p_{uncorr}= 0.037$). Regions related to conflict adaptation (II>CI) did not survive family-wise-error correction (FWE). For completeness, please see **Supplemental Figure 2**.

Supplemental Table S2:

	Absolute change	Standard deviation	SEM
Anxiety symptom changes			
after 3 months	1.8750	8.0282	1.1588
after 6 months	1.0208	7.7170	1.1139
mean across 3&6 months	1.4479	6.9583	1.0043
Depression symptom changes			
after 3 months	0.9583	3.6259	0.5234
after 6 months	0.8750	3.4741	0.5014
mean across 3&6 months	0.9167	3.1696	0.4575

Mean absolute anxiety and depression symptom changes across 48 participants. SEM = standard error of the mean.

Supplemental Table S3:

	Prior internship	After 3 months	After 6 months
Anxiety (clinically relevant cut-off STAI = 40)			
Number of participants above cut-off	9	14	16
Percentage of participants above cut-off	18.75	29.17	33.33
Depression (clinically relevant cut-off PHQ = 10)			
Number of participants above cut-off	2	3	5
Number of participants above cut-off	4.17	6.25	10.42

Number and percentage of participants above clinically relevant cut-offs for anxiety and depression symptom level (N= 48).

Supplemental Table S4: Controlling for adverse events

Predictor	Anxiety Symptom Severity Change (Mean across 3 and 6 months, N = 48) (1)	(2)	(3)	(4)	(5)	(6)	(7)
(Intercept)	1.447917	1.447917	1.447917	1.447917	1.447917	1.447917	1.447917
SE	0.70352	0.713422	0.700669	0.703357	0.711653	0.697828	0.700799
p	0.046137	0.049093	0.0453	0.046088	0.048559	0.044473	0.045338
STAI at T_0	-0.5927	-0.6799	-0.71557	-0.73443	-0.67892	-0.70403	-0.70484
	0.759913	0.766123	0.746227	0.748378	0.763545	0.743462	0.747033
	0.440005	0.380137	0.343359	0.33231	0.379231	0.349343	0.351077
PreTrauma	-0.23772	-0.27104	-0.46067	-0.35552	-0.31105	-0.50935	-0.34473
	0.741307	0.757003	0.757705	0.750458	0.758491	0.758741	0.745788
	0.750121	0.72219	0.546635	0.63826	0.683929	0.505885	0.646419
Behav-CSE	1.530294	1.405472	1.396004	1.488565	1.384966	1.369268	1.434413
	0.739969	0.758263	0.742482	0.741033	0.757618	0.740676	0.740488
	0.045144	0.071191	0.067379	0.051345	0.075007	0.071906	0.059816
LC (CI>II)	1.555266	1.340543	1.631516	1.519396	1.391026	1.700913	1.573512
	0.767049	0.787857	0.771609	0.747496	0.788943	0.773647	0.745549
	**0.0493**	0.09661	**0.040759**	**0.048763**	0.085514	**0.033755**	**0.041112**
Pupil-Dist	0.900454	1.030699	1.054245	1.1316	1.027686	1.049172	1.109088
	0.762795	0.766469	0.75085	0.752295	0.764504	0.747849	0.749788
	0.244787	0.186286	0.168015	0.140386	0.186442	0.168355	0.146919
PPI: LC →Amydala (CI>II)	3.673545	3.741474	3.58908	3.527408	3.70898	3.565374	3.540386
	0.77704	0.787062	0.776721	0.783641	0.78544	0.774416	0.779482
	**2.81E-05**	**2.59E-05**	**3.93E-05**	**5.71E-05**	**2.86E-05**	**4.14E-05**	**5.03E-05**
Adverse Events	0.86563	0.802315	0.834459	0.946016	0.812121	0.842462	0.940417
	0.803425	0.824525	0.801708	0.798715	0.820277	0.797317	0.795836
	0.287746	0.33637	0.304195	0.243236	0.328098	0.297025	0.244314
R^2	0.582406	0.570567	0.585783	0.5826	0.572695	0.589136	0.58563
adj.R^2	0.509327	0.495417	0.513295	0.509555	0.497917	0.517234	0.513115
AIC	295.5251	296.8669	295.1353	295.5028	296.6285	294.7452	295.153
BIC	310.4947	311.8365	310.1049	310.4724	311.5981	309.7148	310.1226
Δ R^2 from (1)							
Δ adj.R^2 from (1)							

Adverse events and mean Anxiety symptom changes are correlated (Pearson-correlation p=0.0058; R=-0.3926). However, the adverse events regressor (orange shading) does not add substantial variance (R^2) or adjusted variance (*adj* R^2), while both LC and LC-Amygdala connectivity remain strong predictors of anxiety symptom changes. Please note: Adverse events and mean depression symptom changes are not correlated (Pearson-correlation p=0.35; R=-0.14).

The models:
(1) original LC
(2) LC_1SD + weighted average
(3) LC_1SD + weighted average + physio-corrected
(4) LC_1SD + weighted average + physio-corrected + un-smoothed data
(5) LC_2SD + weighted average
(6) LC_2SD + weighted average + physio-corrected
(7) LC_2SD + weighted average + physio-corrected + un-smoothed data

Supplemental Table S5: Demographic information

		N	%
Sex	Male	20	41,7
	Female	28	58,3
Relationship-Status	Single	18	37,5
	Married	1	2,1
	In Relationship	28	58,3
	Divorced	1	2,1
Smoking	Yes	2	4,2
	No	46	95,8
Medication	Yes	14	29,2
	No	34	70,8
Drugs/ Neuroenhancers	Yes	3	6,3
	No	45	93,8

	M	SD
Age	24,44	1,999
Alcohol per week (in liter)	3,5213	4,09793

N= Number participants, % = Percentage of participants, M= Mean, SD= Standard deviation.

Supplemental Table S6: Control-analyses statistics and comparison to brainstem regions

Weighted average of smoothed data							
	voxels	CI>II		Anxiety		Depression	
ROIs		*T*	*p*	*R*	*p*	*R*	*p*
LC_2SD	135	3.075	**0.004**	0.37	**0.01**	0.301	**0.038**
LC_1SD	84	2.948	**0.005**	0.357	**0.013**	0.301	**0.038**
MR	77	4.138	**0.001**	0.383	**0.007**	0.275	0.058
DR	200	1.977	0.054	0.182	0.216	0.164	0.266
AMY	2966	1.612	0.114	0.069	0.642	0.123	0.403
SN	1144	1.564	0.124	0.24	0.1	0.1	0.5
VTA	697	0.514	0.61	0.193	0.19	0.138	0.351
Weighted average of physio-corrected smoothed data							
	voxels	CI>II		Anxiety		Depression	
ROIs		*T*	*p*	*R*	*p*	*R*	*p*
LC_2SD	135	2.234	**0.03**	0.407	**0.004**	0.36	**0.012**
LC_1SD	84	2.018	**0.049**	0.397	**0.005**	0.364	**0.011**
MR	77	3.35	**0.002**	0.35	**0.015**	0.271	**0.062**
DR	200	1.503	0.139	0.444	**0.002**	0.31	**0.032**
AMY	2966	1.186	0.242	0.246	0.091	0.249	0.088
SN	1144	2.055	0.045	0.288	0.048	0.254	0.082
VTA	697	1.403	0.167	0.175	0.234	0.19	0.195
Weighted average of physio-corrected unsmoothed data							
	voxels	CI>II		Anxiety		Depression	
ROIs		*T*	*p*	*R*	*p*	*R*	*p*
LC_2SD	135	1.574	0.122	0.388	**0.006**	0.335	**0.02**
LC_1SD	84	1.089	0.282	0.375	**0.009**	0.382	**0.007**
MR	77	2.608	**0.012**	0.173	0.239	0.077	0.605
DR	200	0.748	0.458	0.373	**0.009**	0.071	0.63
AMY	2966	0.946	0.349	0.194	0.186	0.211	0.15
SN	1144	1.607	0.115	0.298	**0.04**	0.199	0.175
VTA	697	1.343	0.186	0.201	0.171	0.209	0.153

CI>II, anxiety- and depression-symptom changes correlation using weighted averaging for LC and brainstem control regions on original data, physio-corrected, unsmoothed and physio-corrected. LC_2SD = Locus coeruleus (2 standard deviations probability map), LC_1SD = Locus coeruleus (1 standard deviation probability map), MR = Medial raphe, DR = Dorsal raphe, AMY = Amygdala, SN = Substantia nigra, VTA = Ventral tegmental area. Significant statistics marked in BOLD.

Supplemental Table S7:

Anxiety Mean Symptom Change (N = 48)<br>LC_1SD, weighted average, physio-corrected, unsmoothed data							
**Predictor**	(1)	(2)	(3)	(4)	(5)	(6)	(7)
(Intercept)	1.447917	1.447917	1.447917	1.447917	1.447917	1.447917	1.447917
SE	0.985622	0.968764	0.91717	0.931947	0.883404	0.706731	0.696852
p	0.148779	0.142156	0.121739	0.127599	0.10868	**0.046924**	**0.043733**
STAI at T_0	-1.931505	-2.112322	-1.732367	-1.777304	-1.441341	-0.666783	
	0.990648	0.98019	0.940674	0.955943	0.916725	0.749815	
	0.057455	**0.036673**	0.072435	0.069848	0.123392	0.379049	
PreTrauma	-0.21733	-0.161508	-0.649743	-0.131699	-0.595935	-0.146715	
	0.990648	0.974324	0.943414	0.9374	0.909048	0.732859	
	0.827345	0.869102	0.494702	0.888926	0.515683	0.842318	
Behav-CSE		1.568115	1.385415	1.256067	1.105188	1.655464	1.591117
		0.976305	0.92727	0.950538	0.903182	0.731013	0.714473
		0.11539	0.142457	0.193347	0.227901	**0.028889**	**0.031236**
LC (CI>II)			2.356064		2.230084	1.599851	1.651312
			0.954762		0.921594	0.748142	0.711878
			**0.017655**		**0.019936**	**0.038488**	**0.025175**
Pupil-Dist				2.032752	1.889098	1.350484	1.426286
				0.953478	0.905761	0.732701	0.717548
				**0.038766**	**0.043124**	0.072543	0.053234
PPI: LC→Amydala (CI>II)						3.77055	3.921255
						0.759851	0.728492
						**0.000013**	**0.000003**
R^2	0.077907	0.128977	0.237027	0.212243	0.308632	**0.56805**	0.559556
adj.R^2	0.036925	0.069589	0.166053	0.138964	0.226326	0.504838	**0.518584**
AIC	323.547572	322.812665	318.455268	319.989645	315.724865	295.147396	**292.082197**
BIC	329.161175	330.29747	327.811273	329.34565	326.952072	308.245803	**301.438202**
Δ R^2 from (1)	0	0.05107	0.15912	0.134336	0.230724	**0.490143**	0.481649
Δ adj.R^2 from (1)	0	0.032664	0.129127	0.102039	0.189401	0.467913	**0.481659**

Multiple Regression Table - Anxiety symptoms.

The table presents the results of seven multiple regression models (different columns) regressing mean anxiety symptom severity change (average symptom changes between 3 and 6 months of real-world stress exposure) on different sets of predictors. Please note, the LC data were extracted from LC-1SD-mask voxels using weighted averaging in the physio-corrected, unsmoothed data to optimize brainstem signals. For each predictor, the first row indicates the standardized beta-estimate, the second row indicates the standard error (SE), and the third row indicates the p-value (p). Bold p-values indicate $p<0.05$.

Red shading indicates information pertaining to variables currently considered gold-standard in predicting anxiety symptom change prior to real-world stress. *STAI at T_0* is the standard anxiety survey obtained before real-world stress and *PreTrauma* refers to adverse events

experienced before the internship. **Purple shading** indicates information pertaining to behavioral and physiological variables related to the emotional-stroop task. *Behav-CSE* refers to the classic behavioral reaction time congruency sequence effect. *LC (CI>II)* refers to the LC upregulation response, i.e.: the neural activity difference comparing CI>II trials measured in the locus coeruleus. *Pupil-Dist* quantifies the impact of the previous trial CI>II difference in pupil dilation on the current trial CI>II difference in pupil dilation (See methods PDD). *PPI: LC→Amydala (CI>II)* refers to the individual strength of functional coupling between the LC and amygdala during the upregulation process. Regressors were z-scored across participants before submission to the multiple regression. Please see the methods section for a detailed description of the quantification of these variables.
Each numbered column represents a different linear model regressing the average anxiety symptom severity change on increasingly more complex combinations of predictors from (1)–(6), while column (7) represents an optimal model with respect to a goodness-of-fit vs. model-complexity trade-off determined using a step-wise regression procedure (Methods). **Blue shading** indicates information regarding Goodness-of-fit. R^2 = variance explained, $adjR^2$ = variance explained adjusted for number of predictors. Bold numbers in these rows indicate the model with the highest R^2 and $adjR^2$. *AIC* = Akaike information criterion, *BIC* = Bayesian information criterion. Bold numbers in these rows indicate the model with the lowest *AIC* and *BIC*. **Grey shading** indicates information regarding improvements of goodness-of-fit with respect to the base-model (1), which does not contain any behavioral and/or physiological conflict adaptation predictors. $\Delta\ R^2$ *from (1)* indicates the differential increase in variance explained. $\Delta\ adj.R^2$ *from (1)* indicates the differential increase in adjusted variance explained.

Supplemental Table S8:

Depression Mean Symptom Change (N = 48)<br>LC_1SD, weighted average, physio-corrected, unsmoothed data							
**Predictor**	(1)	(2)	(3)	(4)	(5)	(6)	(7)
(Intercept)	0.916667	0.916667	0.916667	0.916667	0.916667	0.916667	0.916667
SE	0.400566	0.404596	0.390534	0.398684	0.384917	0.389069	0.382101
p	**0.026861**	**0.028447**	**0.023588**	**0.026412**	**0.021852**	**0.023332**	**0.020643**
STAI at T_0	-1.612391	-1.626606	-1.40522	-1.48889	-1.279165	-1.217592	-1.400051
	0.400748	0.407082	0.407424	0.411219	0.41021	0.454897	0.395291
	**0.000217**	**0.000242**	**0.001272**	**0.00077**	**0.00328**	**0.010643**	**0.000938**
PreTrauma	0.089149	0.095043	-0.089679	0.09785	-0.08239	-0.080723	
	0.400748	0.405176	0.401284	0.399259	0.395542	0.399841	
	0.824966	0.815629	0.824221	0.80756	0.836005	0.841004	
Behav-CSE		0.133937	0.059892	0.034801	-0.032979	-0.022954	
		0.407351	0.39484	0.406653	0.394025	0.399439	
		0.743866	0.880144	0.932198	0.933695	0.954453	
LC (CI>II)			0.854846		0.833528	0.807679	0.83937
			0.415849		0.410113	0.421913	0.395291
			**0.045919**		**0.048464**	0.062575	**0.039251**
Pupil-Dist				0.627527	0.59955	0.587341	
				0.412462	0.398458	0.404462	
				0.135475	0.139891	0.154069	
PPI: LC→Amydala (CI>II)						0.149437	
						0.454111	
						0.743773	
R^2	0.265981	0.26778	0.333299	0.305182	0.3674	**0.369066**	0.332096
adj.R^2	0.233358	0.217856	0.27128	0.240548	0.29209	0.276734	**0.302412**
AIC	237.109836	238.992042	236.49255	238.475324	235.972391	237.845778	**232.579049**
BIC	242.723439	246.476846	245.848555	247.831329	247.199597	250.944185	**238.192652**
Δ R^2 from (1)	0	0.001799	0.067318	0.039202	0.101419	**0.103086**	0.066116
Δ adj.R^2 from (1)	0	-0.015502	0.037923	0.00719	0.058733	0.043377	**0.069054**

Multiple Regression Table - Depression symptoms.
The table presents the results of seven multiple regression models (different columns) regressing mean depression symptom severity change (average symptom changes between 3 and 6 months of real-world stress exposure) on different sets of predictors. Please note, the LC data were extracted from LC-1SD-mask voxels using weighted averaging in the physio-corrected, unsmoothed data to optimize brainstem signals. For each predictor the first row indicates the standardized beta-estimate, the second row indicates the standard error (SE) and the third row indicates the p-value (p). Bold p-values indicate p<0.05. The predictors and goodness-of-fit indices are identical with those used and explained in Supplemental Table S7.

Supplemental Table S9:

Anxiety Symptom Severity Change (Mean across 3 and 6 months, N = 48)							
**Predictor**	(1)	(2)	(3)	(4)	(5)	(6)	(7)
(Intercept)	1.447917	1.447917	1.447917	1.447917	1.447917	1.447917	1.447917
SE	0.985622	0.968764	0.916839	0.931947	0.897097	0.704899	0.702799
p	0.148779	0.142156	0.121608	0.127599	0.114016	**0.046381**	**0.045322**
STAI at T_0	-1.93151	-2.11232	-1.5135	-1.7773	-1.33878	-0.51679	
	0.990648	0.98019	0.958689	0.955943	0.943615	0.758122	
	0.057455	**0.036673**	0.12173	0.069848	0.163341	0.499283	
PreTrauma	-0.21733	-0.16151	-0.50337	-0.1317	-0.42859	-0.04762	
	0.990648	0.974324	0.93239	0.9374	0.913364	0.721413	
	0.827345	0.869102	0.592073	0.888926	0.641326	0.947693	
Behav-CSE		1.568115	1.34862	1.256067	1.135173	1.681278	1.787169
		0.976305	0.928223	0.950538	0.916804	0.728003	0.711719
		0.11539	0.153509	0.193347	0.222524	**0.026032**	**0.015784**
LC (CI>II)			2.382247		2.025124	1.669419	2.014123
			0.962575		0.964808	0.761185	0.713562
			**0.017349**		**0.041869**	**0.034022**	**0.007125**
Pupil-Dist				2.032752	1.604789	1.081084	
				0.953478	0.940197	0.745601	
				**0.038766**	0.095231	0.15468	
PPI: LC→Amydala (CI>II)						3.898553	4.180129
						0.749917	0.719283
						**5.91E-06**	**6.38E-07**
R^2	0.077907	0.128977	0.237577	0.212243	0.287033	**0.570287**	0.541588
adj.R^2	0.036925	0.069589	0.166654	0.138964	0.202156	0.507402	**0.510332**
AIC	323.5476	322.8127	318.4206	319.9896	317.2014	294.8982	**292.0015**
BIC	329.1612	330.2975	327.7766	329.3456	328.4287	307.9966	**299.4863**
Δ R^2 from (1)	0	0.05107	0.15967	0.134336	0.209126	**0.49238**	0.463681
Δ adj.R^2 from (1)	0	0.032664	0.129729	0.102039	0.165231	0.470477	**0.473407**

Multiple Regression Table - Anxiety symptoms.

The table presents the results of seven multiple regression models (different columns) regressing mean anxiety symptom severity change (average symptom changes between 3 and 6 months of real-world stress exposure) on different sets of predictors. Please note, the LC data were extracted without optimizing brainstem signal procedures**.** For each predictor, the first row indicates the standardized beta-estimate, the second row indicates the standard error (SE), and the third row indicates the p-value (p). Bold p-values indicate $p<0.05$.

Red shading indicates information pertaining to variables currently considered gold-standard in predicting anxiety symptom change prior to real-world stress. *STAI at T_0* is the standard anxiety survey obtained before real-world stress and *PreTrauma* refers to adverse events

experienced before the internship. **Purple shading** indicates information pertaining to behavioral and physiological variables related to the emotional-stroop task. *Behav-CSE* refers to the classic behavioral reaction time congruency sequence effect. *LC (CI>II)* refers to the LC upregulation response, i.e.: the neural activity difference comparing CI>II trials measured in the locus coeruleus. *Pupil-Dist* quantifies the impact of the previous trial CI>II difference in pupil dilation on the current trial CI>II difference in pupil dilation (See methods PDD). *PPI: LC→Amydala (CI>II)* refers to the individual strength of functional coupling between the LC and amygdala during the upregulation process. Regressors were z-scored across participants before submission to the multiple regression. Please see the methods section for a detailed description of the quantification of these variables.

Each numbered column represents a different linear model regressing the average anxiety symptom severity change on increasingly more complex combinations of predictors from (1)–(6), while column (7) represents an optimal model with respect to a goodness-of-fit vs. model-complexity trade-off determined using a step-wise regression procedure (Methods). **Blue shading** indicates information regarding Goodness-of-fit. R^2 = variance explained, $adjR^2$ = variance explained adjusted for number of predictors. Bold numbers in these rows indicate the model with the highest R^2 and $adjR^2$. *AIC* = Akaike information criterion, *BIC* = Bayesian information criterion. Bold numbers in these rows indicate the model with the lowest *AIC* and *BIC*. **Grey shading** indicates information regarding improvements of goodness-of-fit with respect to the base-model (1), which does not contain any behavioral and/or physiological conflict adaptation predictors. ΔR^2 *from (1)* indicates the differential increase in variance explained. $\Delta adj.R^2$ *from (1)* indicates the differential increase in adjusted variance explained.

Supplemental Table S10:

Depression Symptom Severity Change (Mean across 3 and 6 months, N = 48)							
**Predictor**	(1)	(2)	(3)	(4)	(5)	(6)	(7)
(Intercept)	0.916667	0.916667	0.916667	0.916667	0.916667	0.916667	0.916667
SE	0.400566	0.404596	0.381934	0.398684	0.380485	0.383559	0.373525
p	**0.026861**	**0.028447**	**0.020794**	**0.026412**	**0.02045**	**0.021533**	**0.018058**
PHQ at T_0	-1.61239	-1.62661	-1.22195	-1.48889	-1.15474	-1.04453	-1.22108
	0.400748	0.407082	0.416355	0.411219	0.418857	0.463821	0.40289
	**0.000217**	**0.000242**	**0.005337**	**0.00077**	**0.008599**	**0.029742**	**0.004035**
PreTrauma	0.089149	0.095043	-0.07256	0.09785	-0.0564	-0.06071	
	0.400748	0.405176	0.388197	0.399259	0.386979	0.390177	
	0.824966	0.815629	0.852615	0.80756	0.884824	0.877112	
Behav-CSE		0.133937	0.023648	0.034801	-0.03996	-0.02541	
		0.407351	0.387007	0.406653	0.38947	0.393432	
		0.743866	0.951558	0.932198	0.918777	0.948809	
LC (CI>II)			1.061555		0.972289	0.957617	1.05126
			0.420391		0.4259	0.4301	0.40289
			**0.015333**		**0.027563**	**0.031535**	**0.012274**
Pupil-Dist				0.627527	0.461313	0.441682	
				0.412462	0.400312	0.404991	
				0.135475	0.25568	0.281819	
PPI: LC→Amydala (CI>II)						0.252992	
						0.440631	
						0.568998	
R^2	0.265981	0.26778	0.362338	0.305182	0.381882	**0.386813**	0.361741
adj.R^2	0.233358	0.217856	0.303021	0.240548	0.308297	0.297078	**0.333374**
AIC	237.1098	238.992	234.3549	238.4753	234.8607	236.4763	**230.3998**
BIC	242.7234	246.4768	243.7109	247.8313	246.0879	249.5747	**236.0134**
Δ R^2 from (1)	0	0.001799	0.096357	0.039202	0.115902	**0.120832**	0.095761
Δ adj.R^2 from (1)	0	-0.0155	0.069663	0.00719	0.074939	0.06372	**0.100017**

Multiple Regression Table - Depression symptoms.

The table presents the results of seven multiple regression models (different columns) regressing mean depression symptom severity change (average symptom changes between 3 and 6 months of real-world stress exposure) on different sets of predictors. Please note, the LC data were extracted without optimizing brainstem signal procedures**.** For each predictor the first row indicates the standardized beta-estimate, the second row indicates the standard error (SE) and the third row indicates the p-value (p). Bold p-values indicate $p<0.05$. The predictors and goodness-of-fit indices are identical with those used and explained in Supplemental Table S7.

Supplemental Figures

Supplemental Figure S1:

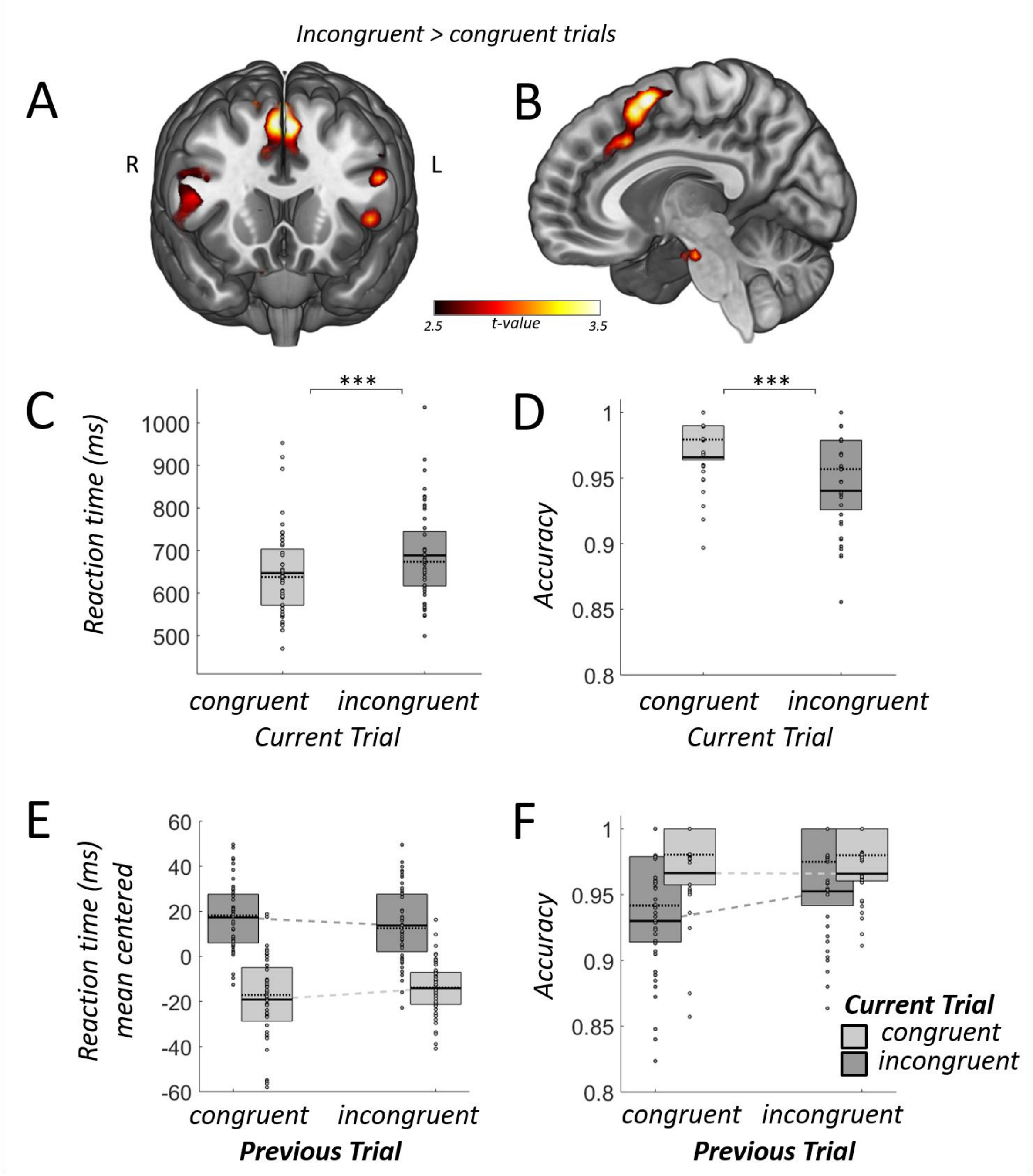

Response conflict (incongruent trials > congruent trials) in dorsomedial prefrontal cortex and replication of classic behavioral conflict results and trial sequence effects.

The imaging analyses focus on incongruent trials because during these trials, the incompatibilities between stimulus dimensions (emotion vs. word) induce response conflict. In order to further confirm

the generality of the response conflict induced by the paradigm, we contrasted incongruent with congruent trials irrespective of trial sequence. Not surprisingly, this contrast revealed the dorsomedial prefrontal cortex (DMPFC, cluster extent = 197, degrees of freedom (df) = 47, nonparametric P(FWE) = 0.032, X/Y/Z: -7/13/55), a region strongly associated with response conflict in several prior studies [10,23,24]. **(A)** Coronal view **(B)** sagittal view. L = left hemisphere, R= right hemisphere. Standard FWE-corrected for a cluster-level threshhold of $p<0.05$ (generated with an initial cluster-forming threshold of T(1,47)=3.277, equivalent to p=0.001). DMPFC, T_{47} = 4.62; p = 0.016^{FWE}, X/Y/Z: -2/11/60. This statistical analysis and its result are comparable to previous seminal studies [10,23,24].

(C-D) Incongruent trials induce significantly more conflict than congruent trials, as indicated by **(C)** increased RTs and **(D)** decreased accuracy. For all boxplots: each dot represents data from a single subject. Top and bottom of boxes indicate 75th and 25th percentile of the underlying distribution respectively. Horizontal lines within boxes indicate the mean (black) and median (dotted).

(E-F) A classic congruency sequence effect was revealed on the **(E)** reaction times and **(F)** accuracy of incongruent compared to congruent trials. The reaction times on incongruent trials show the classic conflict adaptation effect[10], with the presence of an incongruent trial just before the current judgement lead to opposite effects on the reaction times for judgments of incongruent vs congruent stimuli on the present trial.

Supplemental Figure S2:

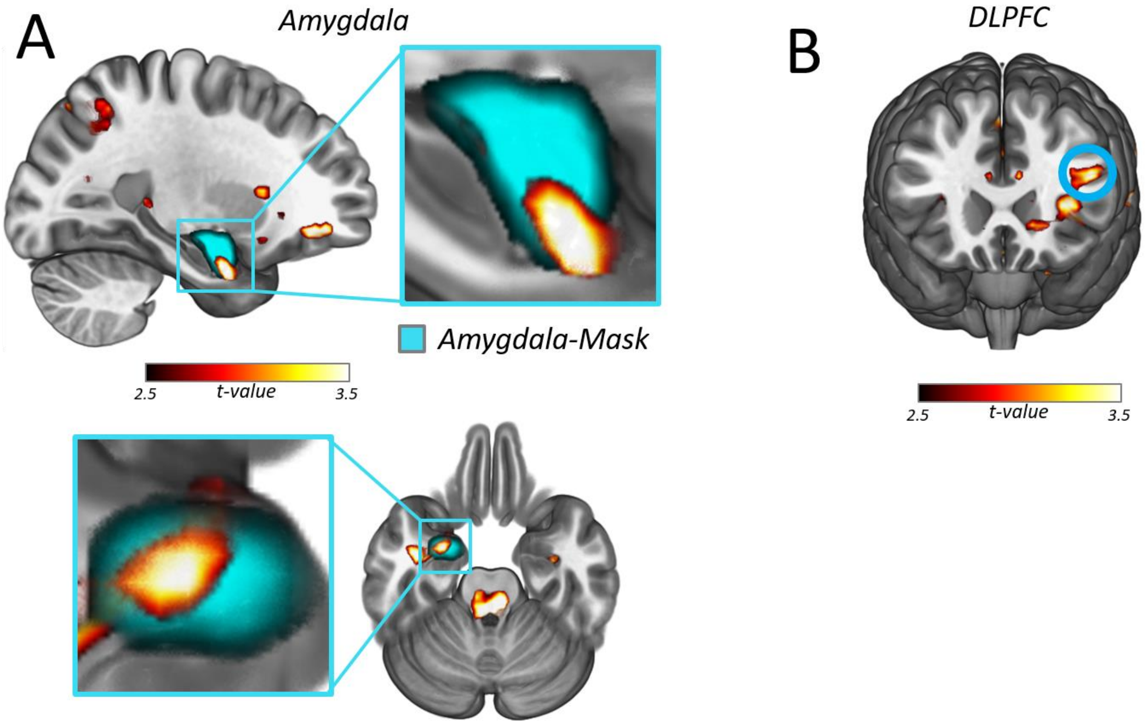

Confirmation of regions previously associated with (CI > II).

In the present data-set we replicate previous upregulation regions identified using the CI>II contrast. **(A)** Amygdala ROI (cyan) is taken from the Harvard-Oxford cortical and subcortical structural atlases as used in the FSL package (https://fsl.fmrib.ox.ac.uk/fsl/fslwiki/Atlases). Left Amygdala XYZ = -27/1/-33, T=3.60, p^{SVC} = 0.045. ^{SVC} indicates small-volume correction. **(B)** DLPFC regions of interest were created using 5mm radius spheres around coordinates provided by Etkin et al. 2006. Left DLPFC: XYZ = -45/21/23, T=3.56, p^{SVC} = 0.006. p_{uncorr} < 0.001. **N.B.**: The right amygdala and right DLPFC (both not shown) failed significance for the contrast (CI>II, Amygdala: XYZ = 21/3/-30, T=2.90, Z=2.77, p^{SVC} = 0.214, p_{uncorr}= 0.003, rDLPFC: XYZ = 43/13/28, T=1.82, Z=1.79, p^{SVC} = 0.176, p_{uncorr}= 0.037).

Supplemental Figure S3:

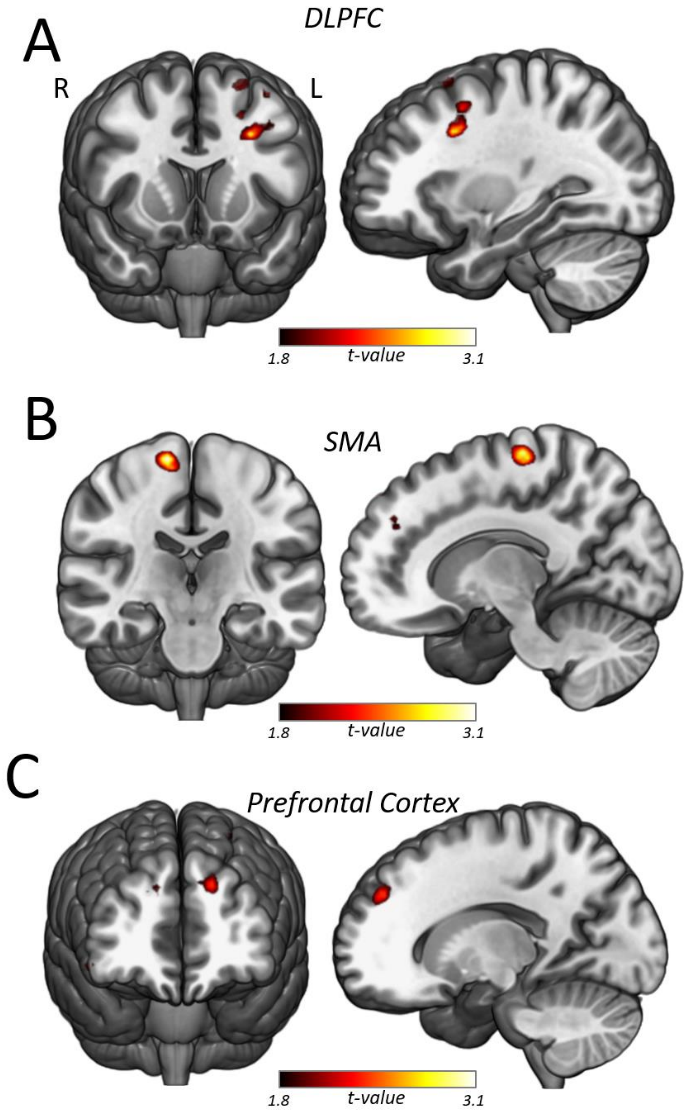

Confirmation of regions previously associated with (II > CI).

We replicate the region most strongly implicated in conflict adaptation (comparisons of II > CI), **(A)** the left DLPFC P(SVC)=0.001, X/Y/Z: -30/13/35) [25,26]. Additional regions: **(B)** supplementary motor area (SMA, P(uncorr)=0.001, X/Y/Z: 13/-22/68) and **(C)** prefrontal cortex (PFC, P(uncorr)=0.01, X/Y/Z: -17/46/33).

Supplemental Figure S4:

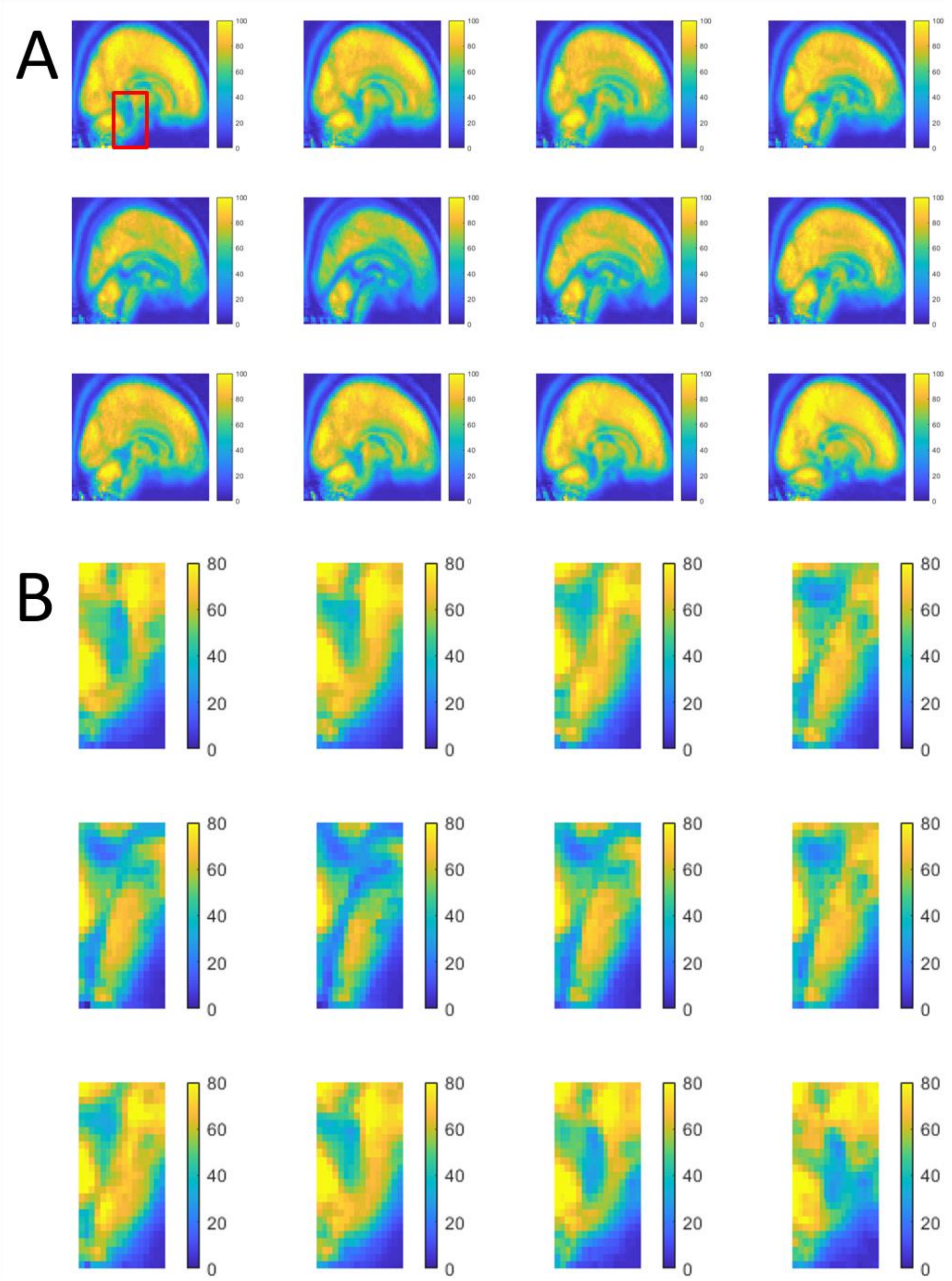

Temporal signal-to-noise ratio across cortex and brainstem.

Temporal signal-to-noise ratio (tSNR) averaged across all 48 participants. (A) Sagittal slices were chosen here to emphasize the difference between signal quality between cortex and brainstem. Color-scale ranges from 0-100. The red rectangle indicates the cutout region displayed in B focusing on the brainstem. (B) tSNR values in sagittal slices of the brainstem. Please not color-scale limits range 0-80 in order to make tSNR contrasts within the brainstem more easily apparent. See next figure for coronal LC-mask comparison and overlay.

Supplemental Figure S5:

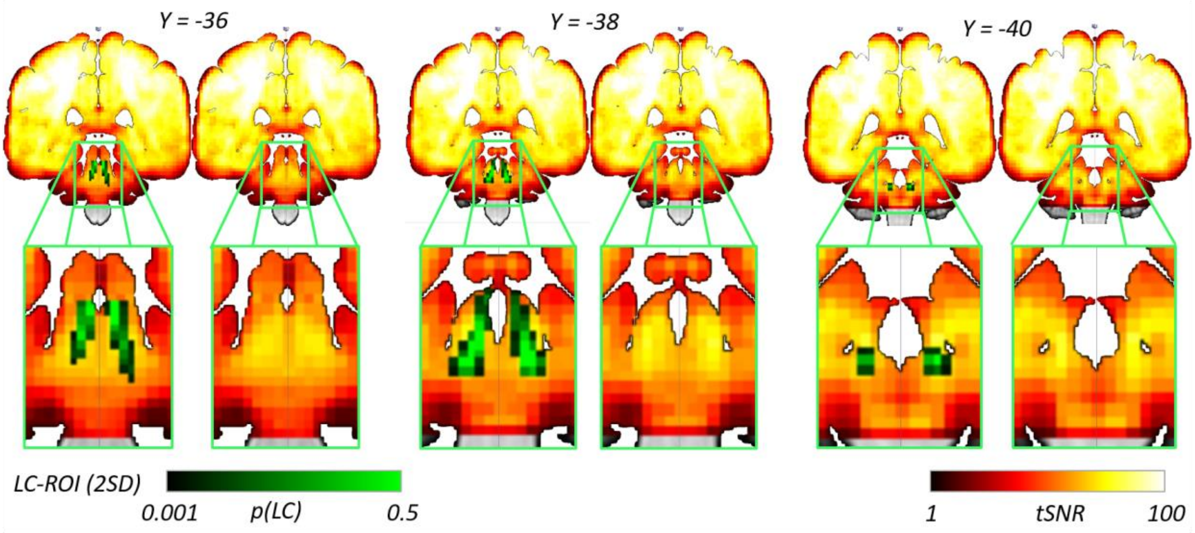

LC-mask and tSNR

Mean temporal signal-to-noise ratio (N= 48) in 3 coronal views slicing through the LC. For easy comparison each different slice-view (indicated by the Y-coordinate above) displays a zoomed view of the same slice containing the probabilistic LC-2SD map on the left (in shades of green) and the identical slice and tSNR overlay on the right.

Supplemental Figure S6:

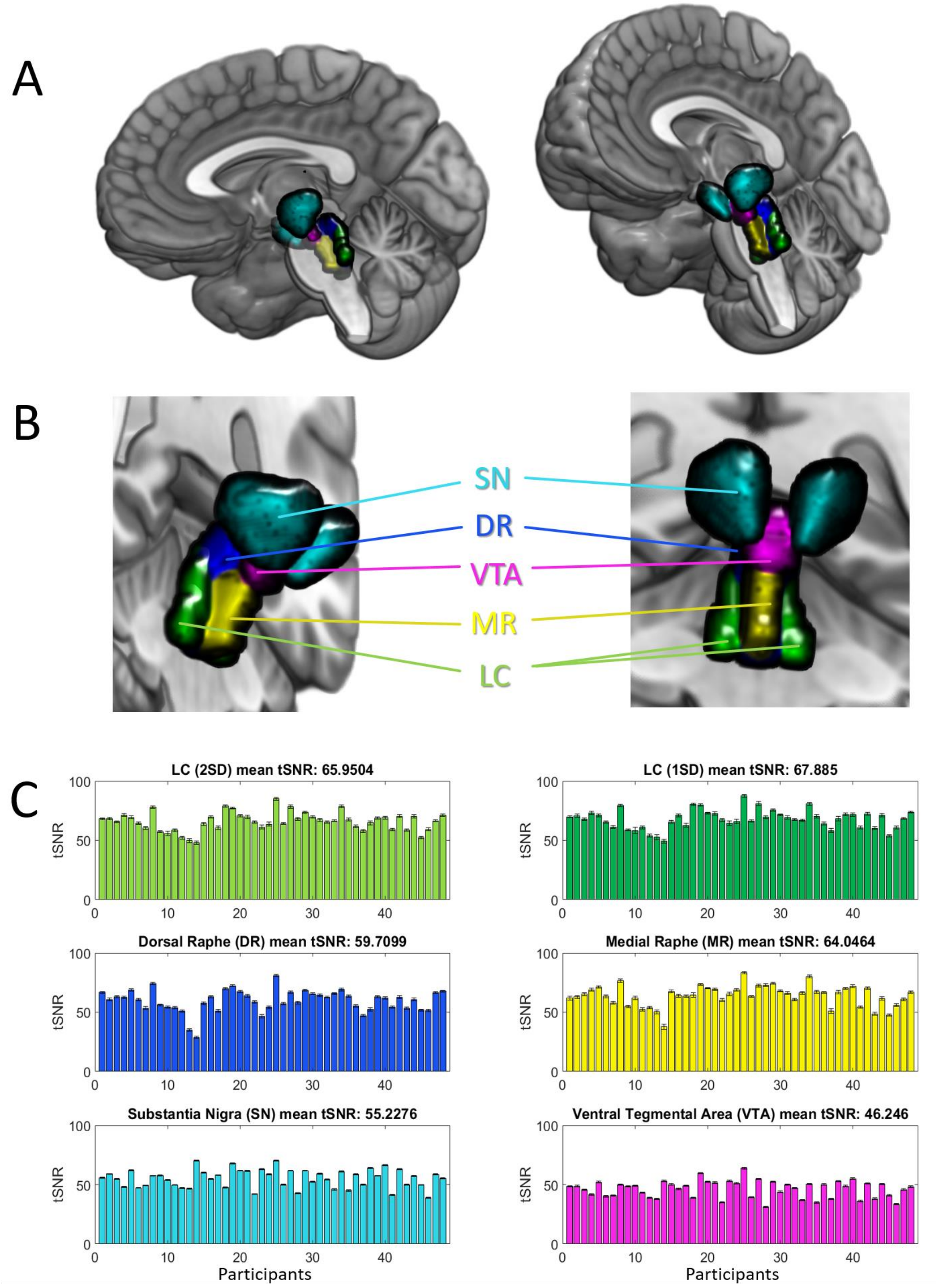

Location of brainstem nuclei and their extracted mean and individual tSNR-values.

(A) Location of brain stem nuclei in reference to the whole brain. (B) Zoomed in version of the brainstem nuclei and their color-code used in C. SN = Substantia nigra, DR = Dorsal raphe, VTA = Ventral tegmental area, MR = Medial raphe, LC = Locus coeruleus. (C) Each panel

contains bar-plots depicting individual tSNR value for each brainstem nuclei and participant (N=48). Each panels title reports the mean tSNR per region.

Supplemental Figure S7:

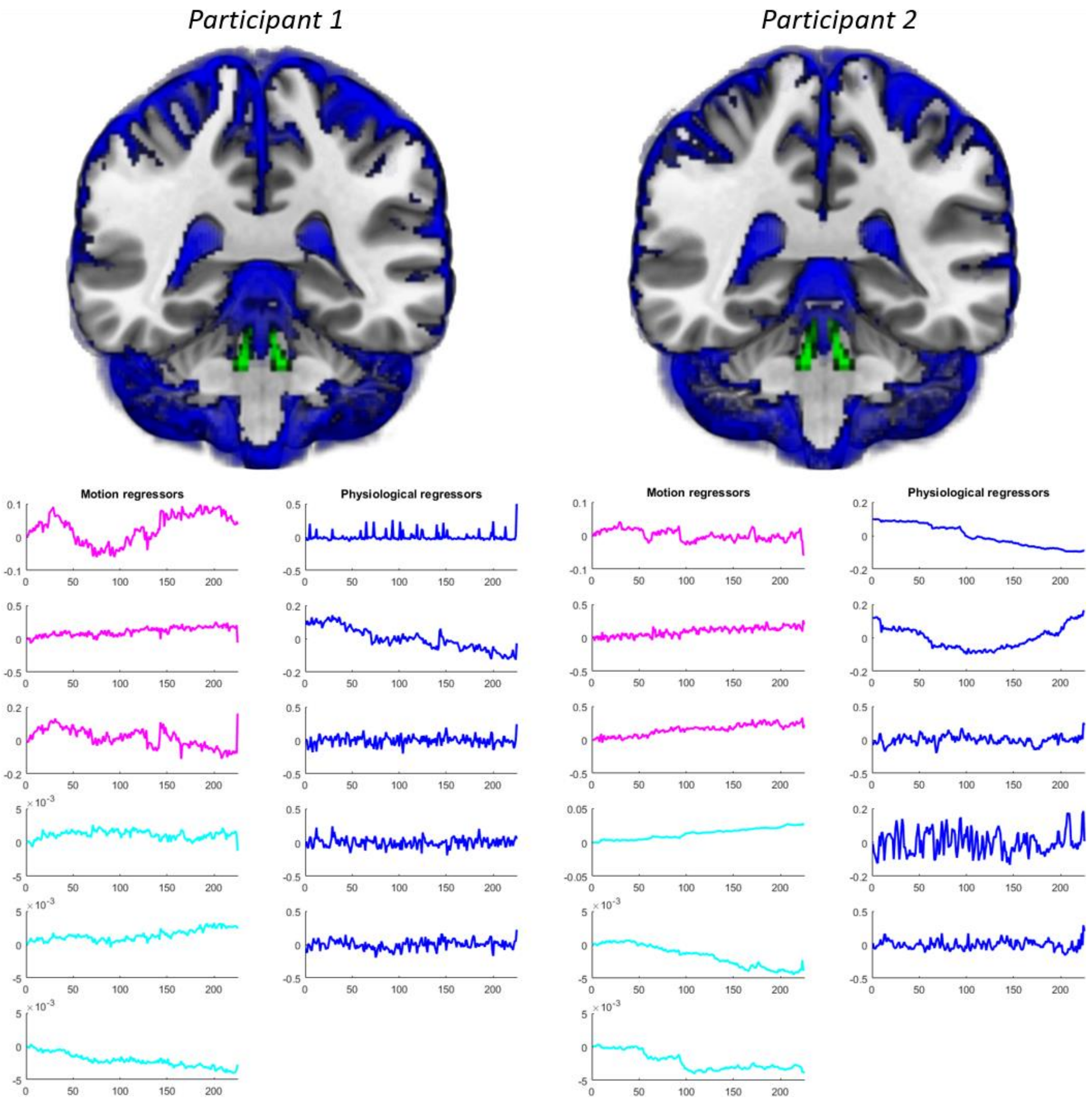

Accounting for physiological noise using individual CSF maps

Physiological noise regressors were derived applying principal component analysis (PCA, Basin et al. 2019) to the time-series in voxels corresponding to each participants' individually segmented CSF probabilistic map as obtained during the 'unified segment' procedure (see Methods). For each subjects GLM (smoothed and unsmoothed data), the first five principal components were added as nuisance regressors along with the six motion regressors obtained during the realignment procedure. The figure illustrates this approach by emphasizing the individuality of the participant-specific CSF tissue map (dark blue) overlayed on a standard brain for the first two subjects. In addition, the motion regressors (translation = magenta, rotation = cyan) and the first five components for each of the two participants (dark blue) are plotted.

Supplemental Figure S8:

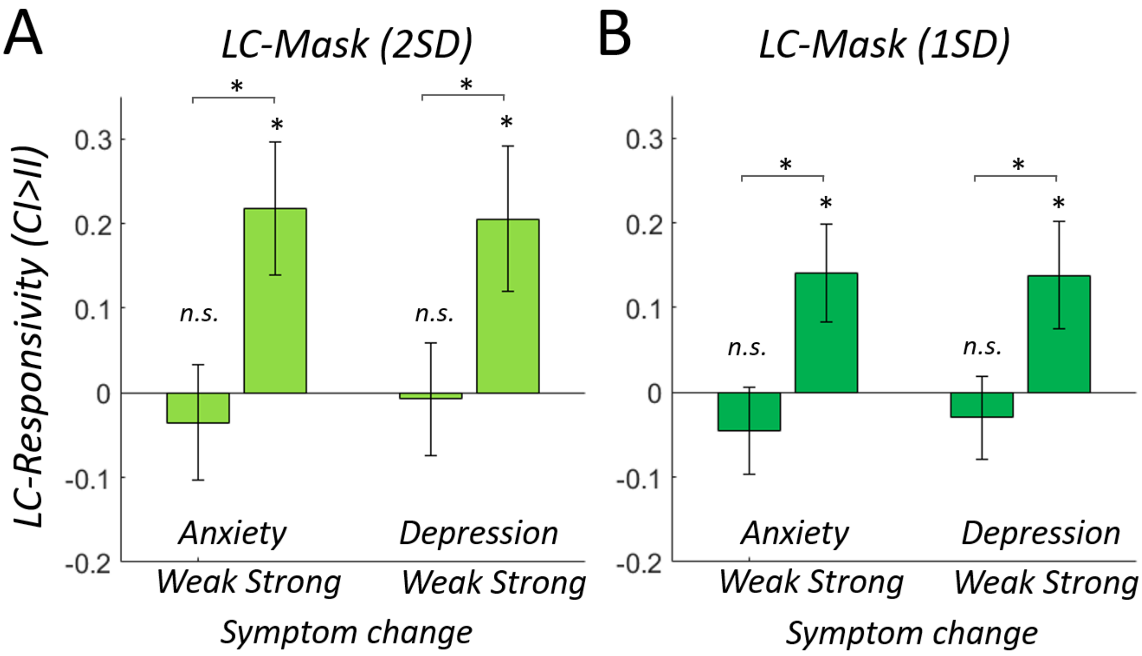

LC_2SD (CI>II)				
	Anxiety		Depression	
	T	*p*	*T*	*p*
High Risk	**2.756**	**0.012**	**2.404**	**0.026**
Low Risk	0.518	0.609	0.104	0.918
High > Low Risk	**2.435**	**0.019**	**1.989**	**0.050**
**LC_1SD (CI>II)**				
	Anxiety		Depression	
	T	*p*	*T*	*p*
High Risk	**2.437**	**0.023**	**2.21**	**0.039**
Low Risk	0.895	0.379	0.611	0.546
High > Low Risk	**2.431**	**0.019**	**2.154**	**0.037**

Strength of LC-responsivity based on subsequent symptom severity change.

Strong changes in anxiety/depression symptoms are accompanied by significantly stronger LC-NE responsivity (CI>II) irrespective of LC-mask. Bar plots show the strength of LC-responsivity (CI>II contrast), extracted from the physiological-noise-controlled, unsmoothed data, as weighted-average of LC-2SD **(A)** and LC-1SD **(B)** mask-voxels, respectively.

Supplemental Figure S9:

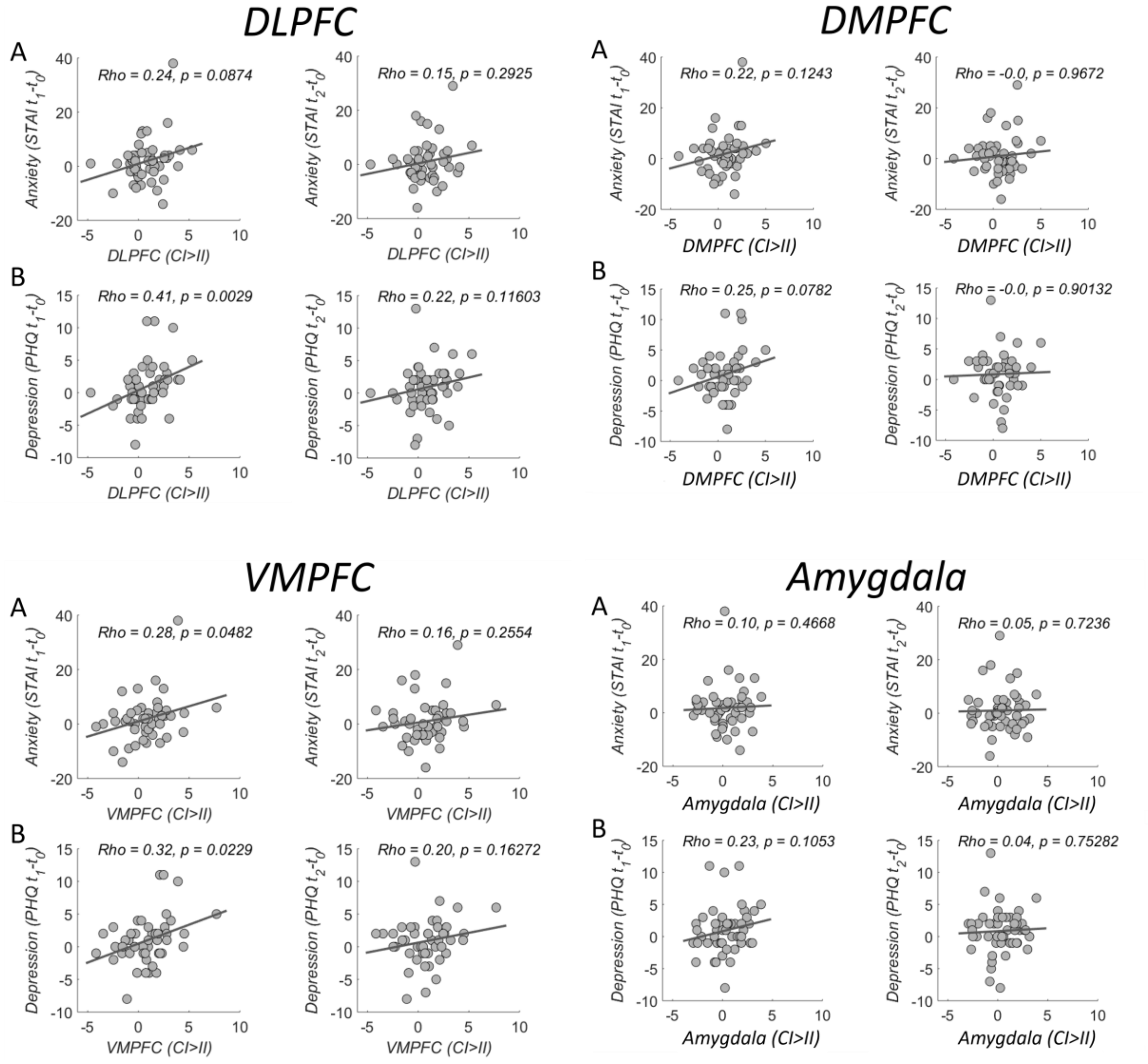

Correlating CI>II regions with anxiety and depression symptom severity change after 3 and 6 months of real-world stress exposure. While the LC upregulation response correlated with anxiety and depression symptoms change after 3 and 6 months of real-world stress exposure (**Main Figure 3**), classic CI>II regions either did not correlate at all (DMPFC, Amygdala), or only for 3 months for both symptom classes (VMPFC) or only depression (DLPFC). Panels A illustrate this for anxiety and panels B for depression.

Supplemental Figure S10:

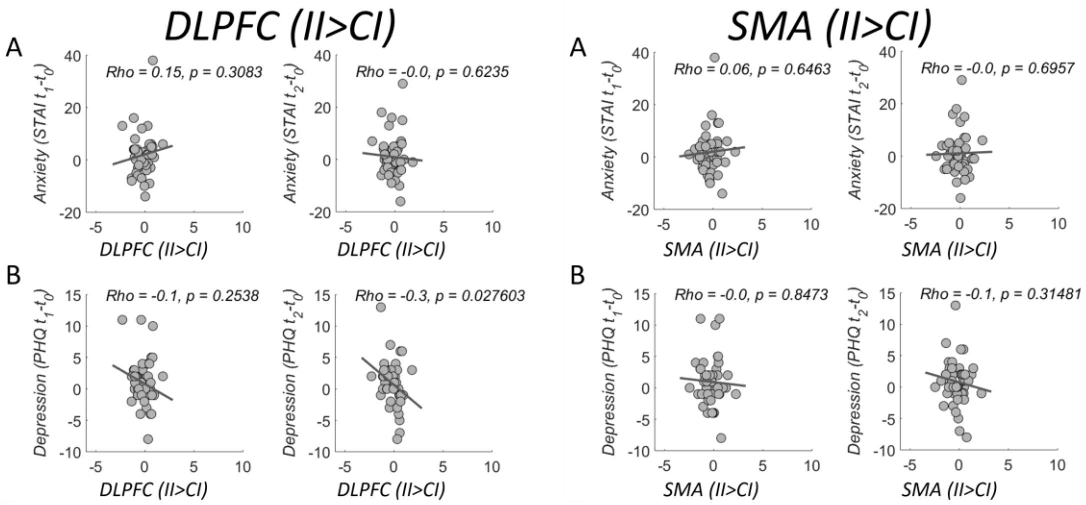

Correlating II>CI regions with anxiety and depression symptom severity change after 3 and 6 months of real-world stress exposure. Neither region shows a consistent relationship of II>CI activity with symptom severity change after 3- or 6-months stress exposure.

Supplemental Figure S11:

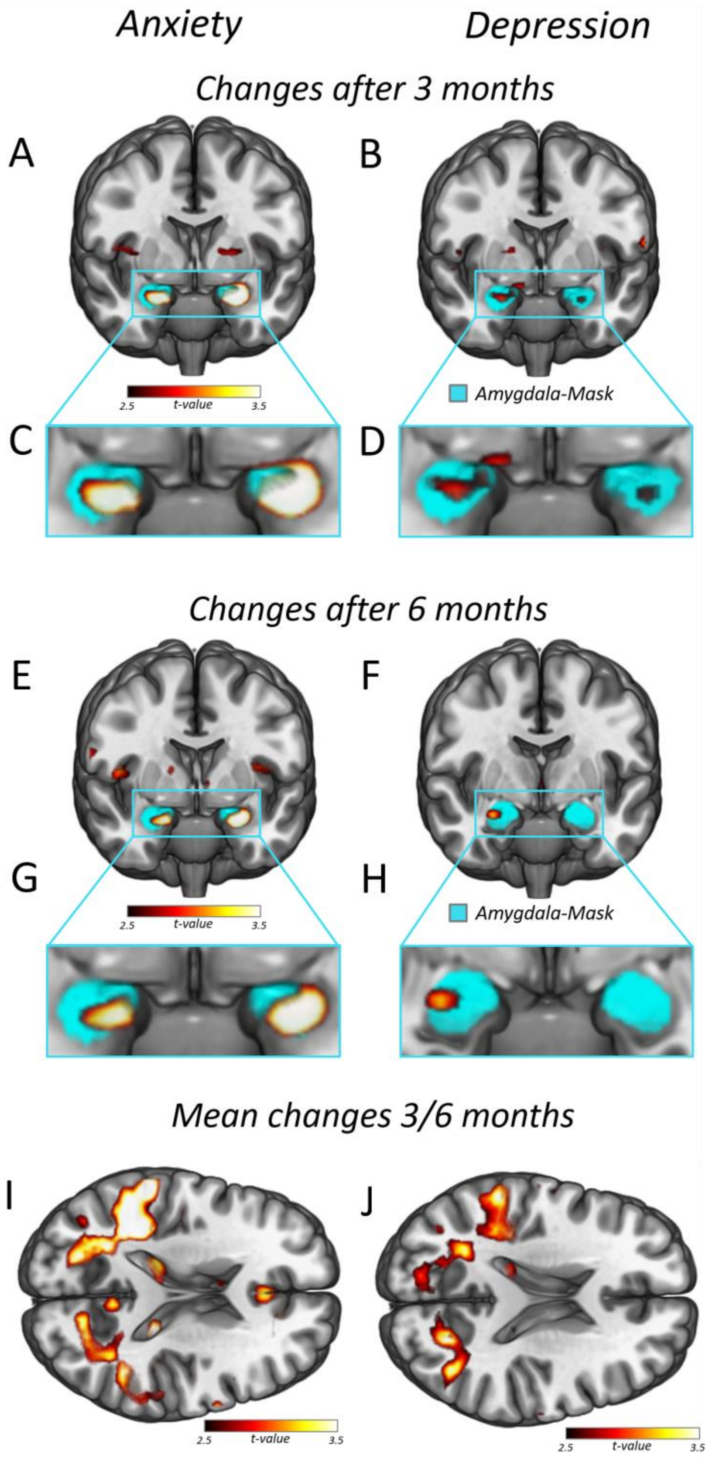

Functional coupling between LC-NE and amygdala during upregulation response relates to symptom changes after 3 and 6 months exposure to real-world stress.

Functional coupling between LC-NE and amygdala during the conflict response relate to individual mean anxiety **(A and C)** and depression **(B and D)** symptom severity changes after 3 months real-world stress exposure. **(E-H)** show the same for the measures acquired after 6

months real-world stress exposure. **(I)** Whole-brain analysis of regions whose functional coupling with the LC-NE exhibits a relationship with mean anxiety changes. Mean symptom changes were defined as the mean between changes after 3 and 6 months. This reveals two symmetric clusters extending bilaterally from IPS to TPJ (Left cluster, p(FWE) = 0.001, IPS: X/Y/Z: -22/-75/25, T = 4.65, Z = 4.19, TPJ: X/Y/Z: -37/-37/18, T = 5.13, Z = 4.54; Right cluster, p(FWE) = 0.004, IPS: X/Y/Z: 23/-72/25, T = 4.40, Z = 4.00, TPJ: X/Y/Z: 41/-50/18, T = 4.62, Z = 4.16). **(J)** These regions were also present when testing this relationship for mean depression changes, but only at an uncorrected level (p<0.001 uncorrected).

Supplemental Figure S12:

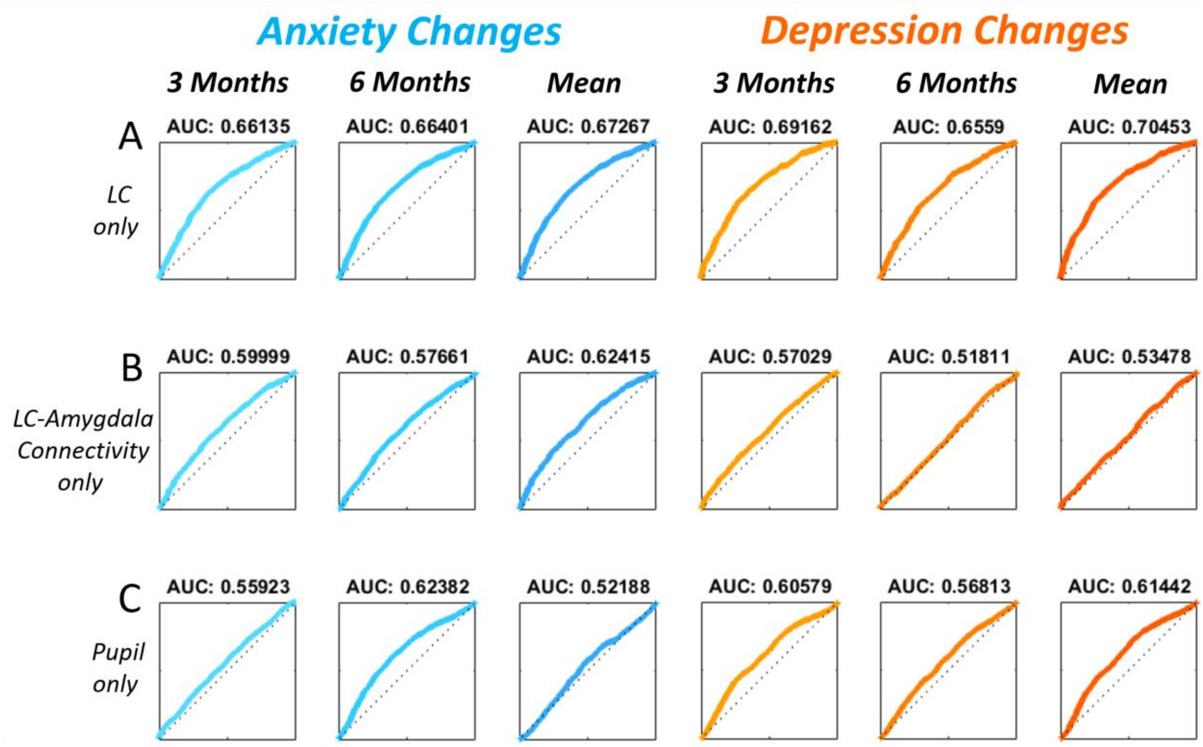

ROC-AUC (Single Factors)

(A) LC responsivity (CI>II) only

(B) LC amygdala-connectivity (CI>II) only

(C) Pupil (CI>II) only

Supplemental Figure S13:

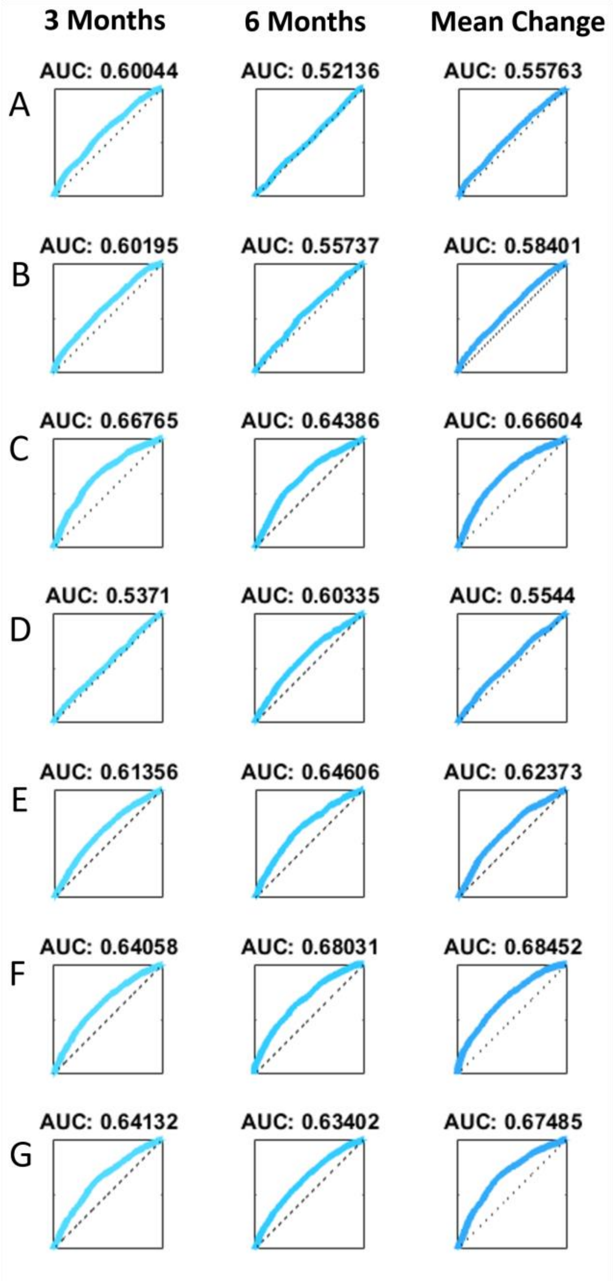

ROC-AUC-Anxiety (Multiple Regressions)

(A) Anxiety at T0 & pre-trauma

(B) Anxiety at T0 & pre-trauma & behavioral score

(C) Anxiety at T0 & pre-trauma & behavioral score & LC

(D) Anxiety at T0 & pre-trauma & behavioral score & Pupil

(E) Anxiety at T0 & pre-trauma & behavioral score & LC & Pupil

(F) Anxiety at T0 & pre-trauma & behavioral score & LC & Pupil & LC→Amygdala

(G) Behavioral score & LC & LC→Amygdala

Supplemental Figure S14:

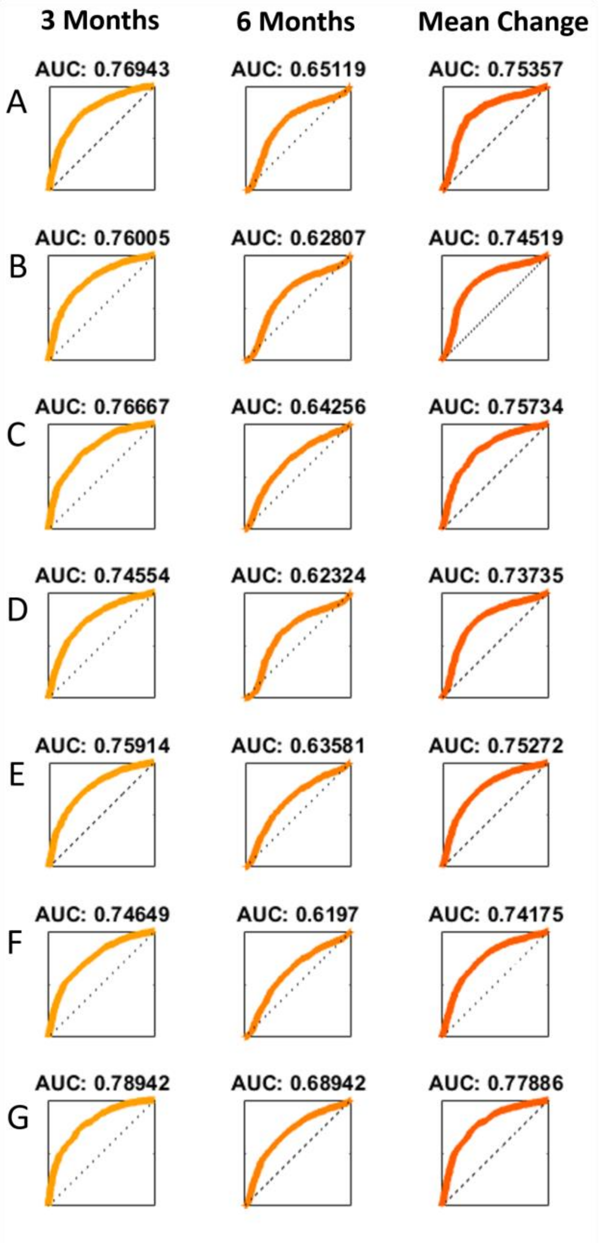

ROC-AUC-Depression (Multiple Regressions)

(A) Depression at T0 & pre-trauma

(B) Depression at T0 & pre-trauma & behavioral score

(C) Depression at T0 & pre-trauma & behavioral score & LC

(D) Depression at T0 & pre-trauma & behavioral score & Pupil

(E) Depression at T0 & pre-trauma & behavioral score & LC & Pupil

(F) Depression at T0 & pre-trauma & behavioral score & LC & Pupil & LC→Amygdala

(G) Depression at T0 & LC

Supplemental References

1 Julian, L. J. Measures of Anxiety. *Arthrit Care Res* **63**, S467-S472, doi:10.1002/acr.20561 (2011).

2 Spielberger, C. D., Gorsuch, R. & Lushene, R. *Manual for the State-Trait Anxiety Inventory: STAI (rev. Ed.)*. (Consulting Psychologists Press, 1983).

3 Spitzer, R. L., Kroenke, K., Williams, J. B. W. & Primary, P. H. Q. Validation and utility of a self-report version of PRIME-MD - The PHQ primary care study. *Jama-J Am Med Assoc* **282**, 1737-1744, doi:DOI 10.1001/jama.282.18.1737 (1999).

4 Kroenke, K., Spitzer, R. L. & Williams, J. B. W. The PHQ-9 - Validity of a brief depression severity measure. *J Gen Intern Med* **16**, 606-613, doi:DOI 10.1046/j.1525-1497.2001.016009606.x (2001).

5 Foa, E. B., Cashman, L., Jaycox, L. & Perry, K. The validation of a self-report measure of posttraumatic stress disorder: The Posttraumatic Diagnostic Scale. *Psychol Assessment* **9**, 445-451, doi:Doi 10.1037//1040-3590.9.4.445 (1997).

6 Mayr, U., Awh, E. & Laurey, P. Conflict adaptation effects in the absence of executive control. *Nature Neuroscience* **6**, 450-452, doi:10.1038/nn1051 (2003).

7 Mayr, U. & Awh, E. The elusive link between conflict and conflict adaptation. *Psychol Res-Psych Fo* **73**, 794-802, doi:10.1007/s00426-008-0191-1 (2009).

8 Monti, J. M., Weintraub, S. & Egner, T. Differential age-related decline in conflict-driven task-set shielding from emotional versus non-emotional distracters. *Neuropsychologia* **48**, 1697-1706, doi:10.1016/j.neuropsychologia.2010.02.017 (2010).

9 Egner, T., Etkin, A., Gale, S. & Hirsch, J. Dissociable neural systems resolve conflict from emotional versus nonemotional distracters. *Cereb Cortex* **18**, 1475-1484, doi:10.1093/cercor/bhm179 (2008).

10 Etkin, A., Egner, T., Peraza, D. M., Kandel, E. R. & Hirsch, J. Resolving emotional conflict: A role for the rostral anterior cingulate cortex in modulating activity in the amygdala. *Neuron* **51**, 871-882, doi:10.1016/j.neuron.2006.07.029 (2006).

11 Ashburner, J. & Friston, K. J. Unified segmentation. *Neuroimage* **26**, 839-851, doi:10.1016/j.neuroimage.2005.02.018 (2005).

12 Valverde, S., Oliver, A., Cabezas, M., Roura, E. & Llado, X. Comparison of 10 Brain Tissue Segmentation Methods Using Revisited IBSR Annotations. *J Magn Reson Imaging* **41**, 93-101, doi:10.1002/jmri.24517 (2015).

13 Dimigen, O., Valsecchi, M., Sommer, W. & Kliegl, R. Human Microsaccade-Related Visual Brain Responses. *Journal of Neuroscience* **29**, 12321-12331, doi:10.1523/Jneurosci.0911-09.2009 (2009).

14 Tse, P. U., Baumgartner, F. J. & Greenlee, M. W. Event-related functional MRI of cortical activity evoked by microsaccades, small visually-guided saccades, and eyeblinks in human visual cortex. *Neuroimage* **49**, 805-816, doi:10.1016/j.neuroimage.2009.07.052 (2010).

15 Eklund, A., Nichols, T. E. & Knutsson, H. Cluster failure: Why fMRI inferences for spatial extent have inflated false-positive rates. *P Natl Acad Sci USA* **113**, 7900-7905, doi:10.1073/pnas.1602413113 (2016).

16 Eklund, A., Knutsson, H. & Nichols, T. E. Cluster failure revisited: Impact of first level design and physiological noise on cluster false positive rates. *Human Brain Mapping* **40**, 2017-2032, doi:10.1002/hbm.24350 (2019).

17 Nichols, T. E. & Holmes, A. P. Nonparametric permutation tests for functional neuroimaging: a primer with examples. *Human Brain Mapping* **15**, 1-25, doi:10.1002/hbm.1058 [pii] (2002).
18 Grueschow, M., Polania, R., Hare, T. A. & Ruff, C. C. Automatic versus Choice-Dependent Value Representations in the Human Brain. *Neuron* **85**, 874-885, doi:10.1016/j.neuron.2014.12.054 (2015).
19 Winkler, A. M., Ridgway, G. R., Webster, M. A., Smith, S. M. & Nichols, T. E. Permutation inference for the general linear model. *Neuroimage* **92**, 381-397, doi:10.1016/j.neuroimage.2014.01.060
S1053-8119(14)00091-3 [pii] (2014).
20 Keren, N. I., Lozar, C. T., Harris, K. C., Morgan, P. S. & Eckert, M. A. In vivo mapping of the human locus coeruleus. *Neuroimage* **47**, 1261-1267, doi:10.1016/j.neuroimage.2009.06.012 (2009).
21 Egner, T. Congruency sequence effects and cognitive control. *Cogn Affect Behav Ne* **7**, 380-390, doi:Doi 10.3758/Cabn.7.4.380 (2007).
22 Botvinick, M. M., Braver, T. S., Barch, D. M., Carter, C. S. & Cohen, J. D. Conflict monitoring and cognitive control. *Psychological Review* **108**, 624-652, doi:10.1037//0033-295X.108.3.624 (2001).
23 Kerns, J. G. *et al.* Anterior Cingulate conflict monitoring and adjustments in control. *Science* **303**, 1023-1026, doi:DOI 10.1126/science.1089910 (2004).
24 Botvinick, M. M., Cohen, J. D. & Carter, C. S. Conflict monitoring and anterior cingulate cortex: an update. *Trends in Cognitive Sciences* **8**, 539-546, doi:10.1016/j.tics.2004.10.003 (2004).
25 Muhle-Karbe, P. S., Jiang, J. & Egner, T. Causal Evidence for Learning-Dependent Frontal Lobe Contributions to Cognitive Control. *Journal of Neuroscience* **38**, 962-973, doi:10.1523/JNEUROSCI.1467-17.2017
JNEUROSCI.1467-17.2017 [pii] (2018).
26 Derrfuss, J., Brass, M., Neumann, J. & von Cramon, D. Y. Involvement of the inferior frontal junction in cognitive control: meta-analyses of switching and Stroop studies. *Hum Brain Mapp* **25**, 22-34, doi:10.1002/hbm.20127 (2005).
