# Supplementary material for: Real-world stress resilience is associated with the responsivity of the locus coeruleus
Source: Nat Commun. 2021 Apr 15;12:2275. doi: 10.1038/s41467-021-22509-1 (PMC8050280; doi:10.1038/s41467-021-22509-1)
Supplement: Supplementary file 3 — Reporting Summary [file 41467_2021_22509_MOESM3_ESM.pdf]

nature research

Corresponding author(s): Marcus Grueschow

Last updated by author(s): Nov 23, 2020

Reporting Summary

Nature Research wishes to improve the reproducibility of the work that we publish. This form provides structure for consistency and transparency in reporting. For further information on Nature Research policies, see Authors & Referees and the Editorial Policy Checklist .

Statistics

For all statistical analyses, confirm that the following items are present in the figure legend, table legend, main text, or Methods section.

n/a	Confirmed	
☐	☒	The exact sample size (*n*) for each experimental group/condition, given as a discrete number and unit of measurement
☐	☒	A statement on whether measurements were taken from distinct samples or whether the same sample was measured repeatedly
☐	☒	The statistical test(s) used AND whether they are one- or two-sided<br>*Only common tests should be described solely by name; describe more complex techniques in the Methods section.*
☐	☒	A description of all covariates tested
☐	☒	A description of any assumptions or corrections, such as tests of normality and adjustment for multiple comparisons
☐	☒	A full description of the statistical parameters including central tendency (e.g. means) or other basic estimates (e.g. regression coefficient) AND variation (e.g. standard deviation) or associated estimates of uncertainty (e.g. confidence intervals)
☐	☒	For null hypothesis testing, the test statistic (e.g. *F*, *t*, *r*) with confidence intervals, effect sizes, degrees of freedom and *P* value noted<br>*Give P values as exact values whenever suitable.*
☒	☐	For Bayesian analysis, information on the choice of priors and Markov chain Monte Carlo settings
☒	☐	For hierarchical and complex designs, identification of the appropriate level for tests and full reporting of outcomes
☐	☒	Estimates of effect sizes (e.g. Cohen's *d*, Pearson's *r*), indicating how they were calculated

Our web collection on statistics for biologists contains articles on many of the points above.

Software and code

Policy information about availability of computer code

Data collection	The emotional-stroop task was presented and behavioral data were collected using custom-written code in MATLAB R2015b and the cogent2000 toolbox. The Eyelink toolbox version 1.6 (https://github.com/uzh/edf-converter) was used to collect pupil dilation and eye-movement data with matlab version 2017a.
Data analysis	The MATLAB toolboxes Statistical Parametric Mapping (version SPM8, https://www.fil.ion.ucl.ac.uk/spm/software/spm8/) and Statistical Non-Parametric mapping (http://warwick.ac.uk/snpm) were used to analyze the imaging data. Standard functions contained in the MATLAB statistics toolbox were used to analyze the behavioral data as well as for conducting statistical tests, multiple regressions, and model comparisons.The longitudinal symptom severity data, pupil and fmri roi results was uploaded to a data-sharing repository (https://github.com/mgrues/LC_Stress_GitHubRepository). Pupil and fMRI brainstem analysis code have been uploaded to code-sharing repository (https://github.com/mgrues/LC_Stress_GitHubRepository).

For manuscripts utilizing custom algorithms or software that are central to the research but not yet described in published literature, software must be made available to editors/reviewers. We strongly encourage code deposition in a community repository (e.g. GitHub). See the Nature Research guidelines for submitting code & software for further information.

Data

Policy information about availability of data

All manuscripts must include a data availability statement. This statement should provide the following information, where applicable:

- Accession codes, unique identifiers, or web links for publicly available datasets
- A list of figures that have associated raw data
- A description of any restrictions on data availability

The longitudinal symptom severity data, pupil and fmri roi results were uploaded to a data-sharing repository (https://github.com/mgrues/LC_Stress_GitHubRepository). Source data are provided as Source Data files for all figures (F1-F9).

Field-specific reporting

Please select the one below that is the best fit for your research. If you are not sure, read the appropriate sections before making your selection.

- [x] Life sciences
- [ ] Behavioural & social sciences
- [ ] Ecological, evolutionary & environmental sciences

For a reference copy of the document with all sections, see nature.com/documents/nr-reporting-summary-flat.pdf

Life sciences study design

All studies must disclose on these points even when the disclosure is negative.

Sample size	Fourty-eight human participants (medical students before a stressful emergency internship) with normal or corrected to normal vision and no history of neurological disease or psychiatric disorders were recruited for this experiment. Participation was voluntary, and participants provided written informed consent. The nature of the real-life population (medical students of one year's cohort at UZH) and the recruiting context prevented us from recruiting a different number of participants. No statistical methods were used to pre-determine sample size but our sample size (N=48) is well above to those reported in comparable fMRI publications (see Methods). For instance Etkin et al. Neuron 2006, on which our paradigm and contrasts are based, used 19 participants and Admon et al. PNAS 2009 using similar brain-symptom correlation approaches employ 37 participants. Our sample size of 48 is well above previous studies and gave us enough power to reliably estimate and replicate activity patterns for previously reported contrasts, as well as having enough data to perform out-of-sample prediction tests. Our sample size was sufficient to both estimate a representative random distribution, as well as to obtain reliable estimates of the between-subject variability.
Data exclusions	We recruited medical students prior to their first medical internship. From the cohort of 200 availabe medical students of the University of Zurich per year, 96 expressed an interest to participate. Forty of the 94 medial students initially expressing interest to participate in the study had to be excluded due to the following a-priori criteria: Insufficient German skills, self-reported psychopathology (including depression and anxiety), a scheduled internship in an area with projected low stress exposure (e.g., dermatology), or fMRI safety exclusion criteria (pacemaker or neurostimulator, hearing aid, insulin or pain pump, implants like cochlea implants or prostheses with metallic parts, irremovable ferromagnetic material on or in the body like piercings, metal splitter injuries, metal clips). Additional exclusion criteria constituted claustrophobia, inability to lie in the MRI scanner due to tremor or coughing, recent (<6 months) unhealed tattoo, and pregnancy. Moreover, six participants canceled their participation or did not appear at the appointed time for the baseline interview. The final sample consisted of 48 medical students (n=28 women, mean age = 24 years, SD = 1.99). Following pre-established criteria based for instance on Etkin et al. Neuron 2006 and Egner 2007 CABN, the behavioral analysis excluded error and post-error trials as well as trials exceeding reaction times larger than 2 standard deviations from the individual distribution.
Replication	Symptom severity predictions were conducted using leave-one-subject out and leave-two-subjects out procedures, constituting out-of-sample replication tests that confirm external validity. The prediction analyses based on leave-two-subject-out cross-validation procedure employed significance testing using a permutation test with 1,000 permutations for each possible left out pair combination (possible pairs = 2256). In addition, numerous fMRI findings of previous studies using the same paradigm for other purposes were replicated and comprehensively listed in the supplementary results section with additional text, figures and tables in the supplemental material.
Randomization	Randomized allocation of participants into different experimental groups is not relevant in our longitudinal within-subject design, as we treated data from all participants equally and correlated their individual psychiatric symptom changes after 3 and 6 months to pre-stress neural and pupil data.
Blinding	Blinding was not applicable in this study as we acquired fMRI and pupil data before the psychiatric symptom changes of interest would even be induced by prolonged professional stress. Hence there was no need to sort participants into control and/or experimental groups. Moreover the analysis of the fMRI and pupil data was done independently of the subsequent symptom data analysis.

Reporting for specific materials, systems and methods

We require information from authors about some types of materials, experimental systems and methods used in many studies. Here, indicate whether each material, system or method listed is relevant to your study. If you are not sure if a list item applies to your research, read the appropriate section before selecting a response.

Materials & experimental systems

n/a	Involved in the study	
x		Antibodies
x		Eukaryotic cell lines
x		Palaeontology
x		Animals and other organisms
	x	Human research participants
x		Clinical data

Methods

n/a	Involved in the study	
x		ChIP-seq
x		Flow cytometry
	x	MRI-based neuroimaging

Human research participants

Policy information about studies involving human research participants

Population characteristics	See above; A comprehensive list of demographic information is provided in supplemental table S1.
Recruitment	In our prospective study we acquired fMRI and pupillometry data in a sample of medical students prior to their first medical internship. This cohort was chosen specifically because medical students constitute a typical at-risk population: They have recently been identified as being alarmingly vulnerable to stress-related disorders, presumably due to ample exposure to significant stress and adversity during their medical internships. This was crucial to our project as we aimed to relate fMRI and pupil-data to future changes in stress symptoms. We could expect such changes to occur throughout the first medical intership, as reported previously. Due to potentially milder forms of stress in Swiss medical internships as compared to American internships our participants may have developed less severe symptom changes as observed in prior studies i.e.: Sen et al. 2010, Arch Gen Psychiat.
Ethics oversight	The Cantonal Ethics Committee of Zurich (KEK) approved the study.

Note that full information on the approval of the study protocol must also be provided in the manuscript.

Magnetic resonance imaging

Experimental design

Design type	Event-related
Design specifications	Each participant, completed 2 fMRI runs of the emotional conflict task, each consisting of 120 trials (60 congruent 60 incongruent trials) as well as 20 randomely interspersed null-event trials consisting of no visual stimulation with an inter-trial variability of 3-7 seconds, sampled from a gamma distribution favouring fast SOAs such as 3-4 seconds. Each run lasted approximatly 10 minutes.
Behavioral performance measures	To interrogate behavioral conflict responding, we used multiple linear (for Reaction Time) and logistic (for Choice Accuracy) regressions onto the following trial-wise predictor variables: current trial congruency, previous trial congruency, interaction of current and previous trial congruency, and current-trial emotional valence. Each regression model was fitted independently for each subject; the resulting parameter estimates were standardized and their deviance from 0 was estimated with a two-sided t-test. Statistical behavioral analyses were performed using the glmfit-, ttest-, and corr-functions implemented in the statistics toolbox in matlab. The results are comprehensively reported in the supplemental material.

Acquisition

Imaging type(s)	EPI, MPRAGE, and GRE field maps
Field strength	3T
Sequence & imaging parameters	225 T2*-weighted whole-brain echo planar images using a Philips Achieva 3 T whole-body scanner (Philips Medical Systems, Best, The Netherlands) equipped with an 8-channel Philips sensitivity-encoded (SENSE) head coil. Imaging parameters were: 2600 ms repetition time (TR); 37 slices (transversal, ascending acquisition); 2.6 mm slice thickness; 2.5 mm x 2.5 mm in-plane resolution; 0.65 mm gap; 90° flip angle. Five dummy-image excitations were performed and discarded before functional image acquisition started. Additionally, we acquired a high-resolution T1-weighted 3D fast-field echo structural scan used for image registration during post-processing (sequence parameters: 181 sagittal slices; matrix size: 256 x 256; voxel size: 1 x 1 x 1 mm; TR/TE/TI: 8.3/2.26/181 ms).
Area of acquisition	whole-brain

Diffusion MRI ☐ Used ☒ Not used

Preprocessing

Preprocessing software	Image preprocessing and analysis were conducted using SPM8 (Wellcome Trust Centre for Neuroimaging). Functional images were slice-time corrected (to the middle slice acquisition time) and realigned (accounting for subjects' head motion). Each subjects' T1-weighted structural image was co-registered to the mean functional image and normalized to the standard T1-MNI template using the "Unified Segment" procedure provided by SPM8. The functional images were then normalized to the standard MNI template using the same transformation, spatially resampled to 2.5 mm isotropic voxels, and smoothed using a Gaussian kernel (FWHM, 6mm).
Normalization	Non-linear "Unified Segment" procedure provided by SPM8.
Normalization template	Standard T1-MNI template
Noise and artifact removal	Six motion parameters (obtained during the realignment procedure) were included as regressors of no interest to account for participants' head motion. Eye movements (saccades) and blinks were added as nuisance-regressors. We controlled for physiological noise with nuisance regressors that reflected the time-course within the cerebrospinal

fluid (CSF). The CSF mask was generated for each individual by the non-linear unified segment procedure in SPM12. Time series were extracted for all voxels included in this mask and were submitted to principle component analysis using the matlab function pca.m included in the statistics toolbox (MATLAB, The MathWorks, Inc., Natick, Massachusetts, U.S.). The first five principle components for each participant were used as nuisance regressors in the GLM analysis, alongside the 6 motion regressors.

We ensured predictive relevance and local specificity for the locus coeruleus by comparing anxiety and depression symptom change predictions based on both LC-masks (1SD and 2SD) and several other brainstem nuclei. In addition, we employed a weighted-average data extraction that weighed every voxel's activity with the probability of membership in the ROI assigned to each voxel. These probabilistic maps included the main brainstem nuclei in the vicinity of the LC, i.e.: medial raphe nucleus (MR), dorsal raphe nucleus (DR), and ventral tegmental area (VTA) provided by the Harvard Ascending Arousal atlas available at https://www.martinos.org/resources/aan-atlas. We also compared LC prediction power with the substantia nigra (SN), available at https://www.nitrc.org/projects/atag/ and the amygdala (https://fsl.fmrib.ox.ac.uk/fsl/fslwiki/Atlases). In addition, we repeated all GLMs also for unsmoothed data, since the 6mm smoothing kernel we applied may have smeared the activity between brain-stem nuclei as well as with adjacent CSF.

Volume censoring

Five dummy-image excitations were performed and discarded before functional image acquisition started, otherwise no censoring was applied.

Statistical modeling & inference

Model type and settings

Mass-univariate random effects model and predictive bootstrapping from regions of interest.

Effect(s) tested

The GLM contained four indicator functions placed at the onset of each of the possible trial types, based on current and previous trial congruency (CI, II, IC, & CC). For instance, CI is an incongruent trial (I) preceded by a congruent trial (C) while II represents an incongruent trial (I) preceded by an incongruent trial (I), and so forth. First-level summary statistics were obtained by calculating the single-subject voxel-wise contrasts of incongruent>congruent trials (I>C, quantifying conflict), CI>II trials (quantifying upregulation) as well as II>CI trials (quantifying conflict adaptation).

Specify type of analysis: ☐ Whole brain ☐ ROI-based ☒ Both

Anatomical location(s)

We ensured predictive relevance and local specificity for the locus coeruleus by comparing anxiety and depression symptom change predictions based on both LC-masks (1SD and 2SD) and several other brainstem nuclei. In addition, we employed a weighted-average data extraction that weighed every voxel's activity with the probability of membership in the ROI assigned to each voxel. These probabilistic maps included the main brainstem nuclei in the vicinity of the LC, i.e.: medial raphe nucleus (MR), dorsal raphe nucleus (DR), and ventral tegmental area (VTA) provided by the Harvard Ascending Arousal atlas available at https://www.martinos.org/resources/aan-atlas. We also compared LC prediction power with the substantia nigra (SN), available at https://www.nitrc.org/projects/atag/ and the amygdala (https://fsl.fmrib.ox.ac.uk/fsl/fslwiki/Atlases). DLPFC regions of interest associated with the conflict response (CI>II) were created using 15mm spheres around coordinates provided by Etkin et al. 2006, and DLPFC regions of interest associated with conflict adaptation were created using 15mm sphere around coordinates provided by Muhle-Karbe, et al. 2017.

Statistic type for inference
(See Eklund et al. 2016)

Statistical inference was performed with a random-effects General Linear Model and cluster-level inference based on non-parametric permutation tests and pseudo t-statistics for independent observations within the SnPM framework (http://warwick.ac.uk/snpm).

Correction

The whole-brain FWE-corrected statistical threshold was set to $P < 0.05$ with an initial cluster-defining voxel-level threshold of $T = 3.275$ (equivalent to uncorrected $P < 0.001$). For hypothesis-guided ROI analysis of the LC-NE arousal system, we applied the identical non-parametric statistical procedure as above restricted to a small volume masks, see above.

Models & analysis

n/a	Involved in the study
☐	☒ Functional and/or effective connectivity
☒	☐ Graph analysis
☐	☒ Multivariate modeling or predictive analysis

Functional and/or effective connectivity

We added to our design matrix the BOLD time-series extracted from a 5mm sphere centered on the subject-specific LC peak in the CI>II contrast, determined by LOSO (see below). We also added two interaction terms corresponding to the interactions of the extracted BOLD time-course and the CI and II regressors.

Multivariate modeling and predictive analysis

Leave-one-subject-out (LOSO) procedure. This method derives an unbiased prediction score, as each participant's data is extracted from a sphere (5mm radius) around the group-peak coordinate in the cortical data or weigted averaging for brainstem data of all other participants, excluding the data from the current participant. For each LOSO analysis, the peak was determined by the CI>II statistical contrast map within the locus coeruleus 2SD-mask from Keren et al., 2009.

Leave-two-subject-out (LTSO) procedure. This method allows us to test how precisely a given model predicts which out of two randomly-drawn participants is more resilient, i.e., will develop lower symptom severity changes. We first generated all possible combinations of training- and test-sets: In each training set, we estimated a given model on the data of N-2 participants and predicted the symptom severity

change for the two left-out participants. We then compared the predicted to the true change score for each of the 2556 possible left-out pairs and determined the prediction accuracy by calculating the percent correct predictions across all left-out pair combinations. In order to quantify how often this accuracy would occur by chance, we generated a null distribution of prediction accuracies. To this end, we repeated the model fit and prediction on shuffled symptom severity labels for each left-out pair 1000 times and calculated the obtained accuracy for each pair combination. The reported p-value represents the probability that the the observed accuracy (based on unshuffled data) occurred by chance. Specifically, the 95th percentile of the null distribution is the lower accuracy bound above which all accuracies exceed a 5% probability of having occurred by chance.
